# Sequential structural rearrangements at the PAM-distal site of a type I-F3 CRISPR-Cas effector enabling RNA-guided DNA transposition

**DOI:** 10.1093/nar/gkaf1415

**Published:** 2026-01-06

**Authors:** Kazuki Ishihara, Shunsuke Matsumoto, Christoph Gerle, Chai C Gopalasingam, Hideki Shigematsu, Tsuyoshi Shirai, Tomoyuki Numata

**Affiliations:** Department of Bioscience and Biotechnology, Graduate School of Bioresource and Bioenvironmental Sciences, Kyushu University, Fukuoka 819-0395, Japan; Department of Bioscience and Biotechnology, Graduate School of Bioresource and Bioenvironmental Sciences, Kyushu University, Fukuoka 819-0395, Japan; Life Science Research Infrastructure Group, RIKEN SPring-8 Center, Hyogo 679-5148, Japan; Life Science Research Infrastructure Group, RIKEN SPring-8 Center, Hyogo 679-5148, Japan; Diffraction and Scattering Division, Japan Synchrotron Radiation Research Institute, SPring-8, Hyogo 679-5198, Japan; Department of Bioscience, Nagahama Institute of Bio-Science and Technology, Nagahama 526-0829, Japan; Department of Bioscience and Biotechnology, Graduate School of Bioresource and Bioenvironmental Sciences, Kyushu University, Fukuoka 819-0395, Japan

## Abstract

Some prokaryotes carry CRISPR-associated transposons (CASTs), Tn7-like elements that incorporate genes encoding CRISPR-Cas effectors. CAST insertion is directed by CRISPR-Cas effectors through RNA-guided DNA binding and interactions with transposition-associated proteins. Although efficient sequence-specific DNA integration requires both precise target DNA recognition and coordinated interactions between effectors and transposition-associated proteins, the underlying mechanism remains elusive. Here, we determined three cryo-EM structures of target DNA-bound type I-F3 TniQ-Cascade from *Vibrio parahaemolyticus*, revealing how Cas8/5 recognizes the protospacer adjacent motif (PAM) and identifying a key residue responsible for the cytidine preference at position -2 of the PAM. We revealed mismatch tolerance at the PAM-proximal site. Structural analyses showed that correct base pairing at the PAM-distal site correlates with conformational changes in the Cas8/5 helical bundle and TniQ, bending the DNA to guide its downstream region toward the transposition machinery. Together, these dynamic rearrangements at the PAM-distal region provide insights into the licensing mechanism of type I-F3 CAST transposition and highlight its potential for genome engineering applications.

## Graphical Abstract

**Graphical Abstract:**
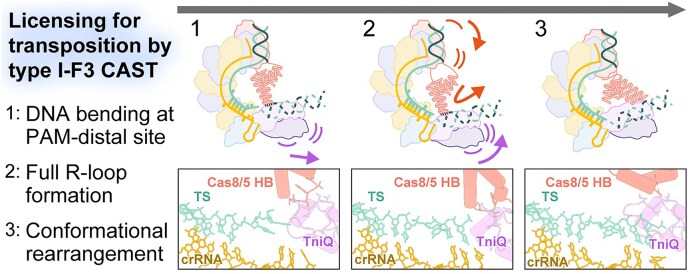

## Introduction

CRISPR-Cas systems provide programmable RNA-guided nuclease activity that serves as an adaptive immune mechanism against invading mobile genetic elements (MGEs) [1–5]. The systems are separated into two major classes, Class 1 and Class 2, which comprise multi-protein CRISPR-Cas effectors and single-protein effectors, respectively. Furthermore, they are divided into seven types (types I–VII) and over 30 subtypes, exhibiting diverse functional and structural characteristics [4–9]. In many systems, the effectors specifically bind to and degrade target MGEs in a sequence-specific manner, thereby suppressing their expression and propagation. Among them, type I systems employ a canonical mechanism in which the CRISPR-Cas effector (Cascade) collaborates with the helicase-nuclease Cas3 to achieve target DNA degradation [10–19]. In contrast, the type I-F3 system and several type I-B variants do not interact with Cas3 [20–22]. Additionally, the type V-K system contains a nuclease-deficient Cas12k protein within its effector and thus lacks DNA-degradation activity [22, 23]. Instead, these systems mediate RNA-guided DNA transposition, wherein the genes encoding CRISPR-Cas effectors are co-opted by Tn7-like transposons to function as CRISPR-associated transposons (CASTs) [20–23]. CAST systems enable site-specific DNA mobility through the coordination of RNA-guided target recognition by CRISPR-Cas effectors and DNA insertion mediated by the transposition-associated proteins TnsA, TnsB, TnsC, and TniQ [20–24].

The type I-F3 Cascade is a multi-subunit complex composed of crRNA, Cas6, Cas7, and Cas8/5, a fusion protein of Cas8 and Cas5 [21]. Cas6 processes the pre-crRNA, transcribed from the CRISPR array, into mature crRNAs, as observed in canonical type I CRISPR-Cas systems [25]. In the type I-F3 CAST system, the crRNA varies depending on the transposition target. For targeting MGEs such as plasmids, typical crRNAs are utilized, whereas atypical crRNAs with divergent repeat sequences are employed for homing into genomic DNA [22, 26]. The type I-F3 Cascade forms a stable complex with TniQ (TniQ-Cascade), which enhances its binding affinity to the target DNA [21, 27]. To date, the cryo-EM structures of TniQ-Cascade from several different species have been reported [27–29]. TniQ is expected to interact with TnsC, a AAA+ ATPase and key regulator of transposition, as demonstrated in the type I-B and V-K CAST systems [30–32]. In the Tn7 transposon system, TnsA and TnsB reportedly cleave the 5′ and 3′ ends of the donor DNA for integration [33–35], while their mechanisms remain unclear in the type I-F3 CAST system, in which TnsC reportedly assembles into a heptamer upon ATP binding [36]. While the precise interactions between the effector and the transposition machinery in the type I-F3 system remain to be elucidated, studies of the V-K CAST system [37] suggested that TnsC serves as a central hub, mediating interactions between the effector and the transposition-associated protein(s) responsible for the excision and integration of donor DNA.

Focusing on target DNA recognition, the type I-F3 CAST system recognizes the 5′-CN-3′ sequence as the protospacer adjacent motif (PAM) [21, 38, 39], exhibiting more promiscuous PAM recognition compared to other systems, in which the types I-B1, I-B2, and V-K recognize 5′-AT-3′, 5′-ATG-3′, and 5′-GTN-3′, respectively [22, 23]. Despite this promiscuity, the on-target rate of the type I-F3 system is ~90%, notably higher than the ∼50% observed for the type V-K system [21, 23], suggesting that type I-F3 could offer improved precision and utility for genome engineering applications. Additionally, the type I-F3 CAST system is distinguished by its unique mismatch tolerance. In canonical CRISPR-Cas systems, a group of several nucleotides downstream of the PAM constitutes the seed sequence, where mismatches between the crRNA and the target DNA are typically not tolerated [40–42]. In contrast, mismatches are often accepted in the PAM-distal region, and significant interference is still detected. In the type I-F3 CAST system, unlike canonical CRISPR-Cas systems, mismatches at PAM-distal sites are not tolerated [21, 36]. Since the PAM-distal region is located near the TnsC binding site, which regulates the transposition site [36], correct base pairings at the PAM-distal region may contribute to the licensing of transposition, although the underlying mechanism remains unclear. Furthermore, several structures have shown that the type I-F3 TniQ-Cascade contains a flexible Cas8/5 helical bundle (HB) domain [28, 29, 43, 44], but its functional roles remain poorly understood. In the *Aeromonas salmonicida* (As) TniQ-Cascade structure, the HB domain apparently stabilized the double-stranded DNA (dsDNA) at the PAM-distal site [27]. Given its structural flexibility, Cas8/5 HB is expected to fulfill additional functional roles.

Here, we determined three distinct structures of the *Vibrio parahaemolyticus* (Vp) TniQ-Cascade, using cryo-EM single-particle analysis. Our findings demonstrated that the correct base pairing between the crRNA and DNA at the PAM-distal region, together with the interactions between TniQ and the Cas8/5 HB, induces the DNA bending necessary for transposition. Furthermore, sequential conformational changes in TniQ and the Cas8/5 HB are required for licensing transposition. Through structural and functional analyses, we elucidated the detailed mechanism of PAM recognition by VpTniQ-Cascade. Our results also revealed partial mismatch tolerance in the seed region during transposition, an unprecedented finding for CAST systems.

## Materials and methods

### Plasmid construction

To construct pETDuet-1-based donor plasmids lacking the T7 promoter, a StuI restriction site was introduced into the upstream region of the T7 promoter for multiple cloning site 1 (MCS1) by site-directed mutagenesis, resulting in the pETDuet-1-StuI plasmid for subsequent experiments.

For the *in vivo* transposition assay, the *tniQ, cas8/5, cas7*, and *cas6* gene operon and the *tnsA, tnsB*, and *tnsC* gene operon were amplified by polymerase chain reaction (PCR), using the genomic DNA of *V. parahaemolyticus* RIMD 2210633 as the template. The *tniQ, cas8/5, cas7*, and *cas6* operon and the *tnsA, tnsB, tnsC* operon were cloned into pCDFDuet-1 (NdeI/XhoI sites) and pCOLADuet-1 (NcoI/XhoI sites), respectively, using an In-Fusion^®^ HD Cloning Kit (TaKaRa). Two BsaI recognition sites and repeat sequences of the CRISPR array were introduced into pCDFDuet-1 (NcoI/NotI sites) harboring the *tniQ, cas8*/*5, cas7*, and *cas6* operon, enabling the facile construction of a co-expression plasmid for any desired crRNAs depending on the experiments.

For the transposition assay in *Escherichia coli* strain BL21(DE3), cargo sequences (a chloramphenicol-resistance gene or a partial DNA fragment of the *tiaS* gene encoding an archaeal tRNA-modifying enzyme) flanked by the right and left ends of VpCAST were synthesized by GeneArt (Thermo Fisher Scientific). These DNA sequences were cloned into pUC18 (EcoRI/HindIII sites) or pETDuet-1-StuI (StuI/XhoI sites), respectively, using an In-Fusion^®^ HD Cloning Kit (TaKaRa). This resulted in the construction of donor plasmids.

For the genome-editing experiment in the methionine-auxotroph *E. coli* strain B834(DE3), the cargo DNA containing the *metE* gene was amplified from the genomic DNA of BL21(DE3) by PCR, and the right and left end sequences of VpCAST were PCR-amplified using the above-mentioned donor plasmid of pETDuet-1-StuI as the template. The resulting three DNA fragments and the pETDuet-1-StuI plasmid, linearized with the StuI and XhoI restriction enzymes, were assembled using a NEBuilder HiFi DNA Assembly Kit (New England Biolabs).

To investigate the mismatch tolerance of TniQ-Cascade, the full-length *hflK–hflC* operon was synthesized by GeneArt (Thermo Fisher Scientific). The *hflK–hflC* operon was PCR-amplified using the synthetic gene fragment as the template, while the pACYC184 vector backbone was amplified by inverse PCR. The resulting DNA fragments were assembled using a NEBuilder HiFi DNA Assembly Kit (New England Biolabs) to construct pACYC184_*hflKC*, which served as the target plasmid for transposition by the VpCAST system. Using pACYC184 as the template, a plasmid (pTarget-32) containing a 32-bp protospacer sequence from *hflK* (nucleotides 655–686) was generated by inverse PCR followed by ligation.

To overproduce VpTniQ-Cascade in *E. coli* cells, the *tniQ, cas8/5, cas7*, and *cas6* operon was cloned into pMAL-c2 (New England Biolabs) at the BamHI and HindIII sites by an In-Fusion^®^ HD Cloning Kit (TaKaRa). To remove the maltose-binding protein (MBP) tag fused to the N-terminus of TniQ, a PreScission Protease recognition site was introduced by site-directed mutagenesis between the coding sequences of MBP and TniQ. The repeat and spacer sequences constituting the first typical crRNA derived from *V. parahaemolyticus* RIMD 2210633 were generated by oligo annealing and cloned into pACYCDuet-1 (NcoI/XhoI sites) by using an In-Fusion^®^ HD Cloning Kit (TaKaRa).

The oligonucleotide sequences used in this study are provided in Supplementary Table S1. The nucleotide sequences of the plasmids used in this study are listed in Supplementary Table S2. The amino acid sequences of each component of TniQ-Cascade and the transposition-associated proteins from *V. parahaemolyticus* RIMD 2210633 are summarized in Supplementary Table S3.

### *In vivo* reconstitution of transposition into *E. coli* BL21(DE3) chromosome

For DNA transposition into the genomic DNA of *E. coli* strain BL21(DE3), the cells were first co-transformed with the plasmids pCOLADuet-1_TnsABC and pUC18_Donor-*cat*, which carried a cargo DNA encoding the chloramphenicol resistance gene. Competent cells were then prepared using the resulting transformants and subsequently further transformed with the plasmid pCDFDuet-1_TniQ-Cascade_Target-*lacZ*, which harbored a spacer sequence targeting *lacZ*. Transformants were plated on LB agar containing 50 μg/ml ampicillin, 50 μg/ml kanamycin, 50 μg/ml streptomycin, and 0.1 mM isopropyl-β-D-thiogalactopyranoside (IPTG), and incubated at 25°C for 39 h. Colonies were resuspended in LB medium, and the optical density at 600 nm (OD_600_) was adjusted to 1.0. The cells in the resulting suspension (1 ml) were collected by centrifugation at 5090 × *g* for 2 min. The pellet was resuspended in 50 μl of water and lysed by heating at 95°C for 10 min. After centrifugation at 5090 × *g* for 5 min, the supernatant containing genomic DNA was collected. This lysate was diluted five-fold and used as the template for PCR to detect transposition. Primers for the positive control were designed to anneal to genomic sequences in both the forward and reverse orientations. Primers for detecting transposition were designed to anneal to both the genomic DNA and the cargo sequence. PCR products were analyzed by electrophoresis on a 1.5% agarose gel, which was stained by ethidium bromide. The locations of transposition sites in the genome were determined by Sanger sequencing.

### Preparation of TniQ-cascade

Streptomycin-resistant *E*. coli strain BL21-CodonPlus(DE3)-RIL competent cells were co-transformed with pMAL-c2_PreScission Protease_TniQ-Cascade and pACYCDuet-1_crRNA1. The cells were cultured in LB medium containing 50 μg/ml ampicillin and 34 μg/ml chloramphenicol at 37°C until the OD_600_ reached ~0.6–1.0, after which IPTG was added to a final concentration of 200 μM. The culture was then incubated at 20°C for 20 h. Cells were harvested by centrifugation at 5480 × *g* for 10 min, followed by 8000 × *g* for 10 min. The cell pellet was resuspended in sonication buffer [20 mM Tris–HCl, pH 7.4, 200 mM NaCl, 1 mM ethylenediaminetetraacetic acid (EDTA), 7 mM 2-mercaptoethanol, 1 mM benzamidine, and 1 mM phenylmethylsulfonyl fluoride (PMSF)] and lysed by sonication. The lysate was then centrifuged at 7600 × *g* for 15 min, followed by 14300 × *g* for 15 min to remove precipitates. The resulting supernatant was applied to amylose resin (New England Biolabs), washed with wash buffer (20 mM Tris–HCl, pH 7.4, 200 mM NaCl, 1 mM EDTA, 7 mM 2-mercaptoethanol), and eluted with elution buffer (20 mM Tris–HCl, pH 7.4, 200 mM NaCl, 1 mM EDTA, 7 mM 2-mercaptoethanol, 10 mM maltose monohydrate) to obtain MBP-tagged TniQ-Cascade. The MBP tag was removed using PreScission Protease (Cytiva) in dialysis buffer (20 mM Tris–HCl, pH 7.4, 200 mM NaCl, 7 mM 2-mercaptoethanol) at 4°C for 20 h. The reaction mixture was then applied to Glutathione Sepharose 4B™ resin (Cytiva) to remove the protease by affinity trapping. The flow-through fraction was diluted to a final NaCl concentration of 100 mM and purified using a HiTrap Heparin HP column (Cytiva), eluted with a linear gradient of 100–1000 mM NaCl in buffer (20 mM Tris–HCl, pH 7.4, 7 mM 2-mercaptoethanol). TniQ-Cascade was further purified by size-exclusion chromatography (SEC) using a Superose 6 Increase 10/300 GL column (Cytiva) in SEC buffer, containing 20 mM Tris–HCl, pH 7.4, 5 mM MgCl_2_, and 1 mM dithiothreitol, with either 100 or 300 mM NaCl.

To prepare the dsDNA-bound TniQ-Cascade, oligonucleotides were annealed in hybridization buffer (20 mM Tris–HCl, pH 7.4, 100 mM NaCl, and 5 mM MgCl_2_) by heating at 95°C for 5 min, followed by gradual cooling to room temperature over 1 h. TniQ-Cascade and dsDNA were mixed at a molar ratio of 1:3, and the resulting mixture was incubated at 37°C for 5 min to reconstitute the complex. The TniQ-Cascade-dsDNA complex was then loaded on a Superose 6 Increase 10/300 GL column (Cytiva) in SEC buffer. The purified complex was concentrated using Amicon Ultra-0.5 30K centrifugal filter units (Merck Millipore).

### Cryo-EM sample preparation and data collection

Grids for cryo-EM analysis of TniQ-Cascade-dsDNA complexes were prepared under various conditions. Differences among preparations included sample concentration, the presence and type of detergent [lauryl maltose neopentyl glycol (LMNG) or CHAPS], and blotting parameters. For full R-loop state 2, grids were prepared without any detergent. Detailed conditions for each structural state are described below.

For the full R-loop state 1 (FR1), TniQ-Cascade-dsDNA (6.5 mg/ml) was supplemented with CHAPS to a final concentration of 8 mM. A 3 μl portion of the sample was applied to a glow-discharged Cu 200-mesh R1.2/1.3 grid (Quantifoil). Using a Vitrobot Mark IV system (Thermo Fisher Scientific), excess liquid was blotted with Whatman Grade 595 Qualitative Filter Papers (110 mm diameter, Cytiva) at 8°C and 100% humidity with a blot force of 0 for 2 s, before the grid was plunge-frozen in liquid ethane (all samples were vitrified in liquid ethane).

For the full R-loop state 2 (FR2), 3 μl of TniQ-Cascade-dsDNA (7.6 mg/ml) without any detergent was applied to a glow-discharged Cu 300 mesh R1.2/1.3 grid (Quantifoil). Blotting was performed at 8°C and 100% humidity with a blot force of 20 for 10 s.

For the partial R-loop state (PR), 3 μl of TniQ-Cascade-dsDNA (2.0 mg/ml) in the presence of 0.001% LMNG was applied to a glow-discharged Cu 200 mesh R1.2/1.3 grid (Quantifoil). Blotting was performed at 8°C and 100% humidity with a blot force of 0 for 2 s.

Data were collected at SPring-8, using a CRYO ARM™ 300 (JEOL) equipped with a K3 camera, operated in the CDS electron-counting mode (Gatan), and an in-column Omega energy filter. YoneoLocr [45] was used for hole detection. Automated data acquisition was performed with SerialEM [46] at a nominal magnification of 60 000×, corresponding to a pixel size of 0.752 Å. Movies were collected with a total electron exposure of 50 e⁻/Å² over 50 frames, using a target defocus range of −1.4 μm to −1.6 μm, with a 5 × 5 beam-image shift pattern.

### Image processing

Single-particle analysis workflows for TniQ-Cascade-dsDNA in the FR1, FR2, and PR states are shown in Supplementary Figs S1, S2, and S3, respectively.

For the FR1 state, 8388 movies were imported into RELION-4.0.1 [47] for motion correction. Motion-corrected micrographs were transferred to CryoSPARC™ v4.6.1 [48] for contrast transfer function (CTF) estimation, and micrographs with CTF fit resolutions worse than 4 Å were excluded. Initial particle picking from 50 micrographs was performed using Blob Picker (diameter 180–230 Å). Particles with contaminants were excluded, and the remaining particles were extracted with a box size of 460 pixels and Fourier-cropped to 80 pixels. Using the 2D class averages as templates, particle picking was conducted across all micrographs (with CTF fit resolutions <4 Å), with a particle diameter of 230 Å. Subsequently, the extracted particles were subjected to 2D classification. Initial 3D models were generated by Ab-Initio Reconstruction in CryoSPARC™ with *K* = 3, using an initial resolution of 35 Å and a maximum resolution of 12 Å. Using these references, contamination was removed through heterogeneous refinement with a box size of 128 pixels. The major class was refined using Non-Uniform Refinement, after particles were re-extracted at 460 pixels. Particles optimized by CTF refinement were then imported into RELION-4.0.1 for Bayesian polishing. Particles were re-imported into CryoSPARC™ v4.6.1 and classified by 3D classification without alignment (*K* = 10). The class in which Cas8/5 HB was most clearly visualized was refined using Non-Uniform Refinement. The resolution was determined based on the gold-standard Fourier shell correlation (FSC) criterion at 0.143 [49]. Local resolution was computed using the Local Resolution Estimation tool. Regions including Cas8/5 HB, TniQ, Cas7.1, and Cas6 were refined by Local Refinement.

For the FR2 state, a total of 7926 movies were motion-corrected using RELION-3.1.2 [50], and corrected micrographs were imported to CryoSPARC™ v4.2.1 [48]. Micrographs with CTF fit resolutions worse than 4 Å were discarded. Particle picking was initially performed on a subset of 50 micrographs, followed by Template Picker, extraction (box size: 460 pixels, Fourier-cropped to 80 pixels), 2D classification, and ab-initio reconstruction (*K* = 5) with an initial resolution of 35 Å and a maximum resolution of 12 Å. The TniQ-bound class was selected and refined by Non-Uniform Refinement with re-extracted particles at 460 pixels. Subsequently, 3D classification without alignment (*K* = 5) was performed, and particles from the class in which the HB was detected were imported into RELION-3.1.3 for CTF refinement and polishing. After signal subtraction, structural heterogeneity was assessed by focused 3D classification without alignment (*K* = 3, *T* = 30), in which the focused regions included Cas8/5 HB, two TniQ molecules, Cas7.1, and Cas6. The original particles were re-imported into CryoSPARC™ v4.2.1 for Non-Uniform Refinement. Resolution was determined using the gold-standard FSC criterion of 0.143 [49], and the local resolution was estimated. Local Refinement was applied to the Cas8/5 HB, TniQ, Cas7.1, and Cas6 regions, and focused maps were sharpened using DeepEMhancer [51].

For the PR state, 4906 movies were imported into RELION-4.0.1 [47], and beam-induced motion was corrected. Motion-corrected micrographs were imported into CryoSPARC™ v4.2.1 [48], and CTF parameters were estimated. Micrographs with a CTF fit resolution worse than 4 Å were removed, and initial particle picking was performed using Blob Picker in CryoSPARC™, targeting circular and elliptical particles with a minimum diameter of 180 Å and a maximum diameter of 230 Å from 50 micrographs, followed by Template Picker, extraction (box size: 460 pixels, Fourier-cropped to 80 pixels), 2D classification, and ab-initio reconstruction (*K* = 5) with an initial resolution of 35 Å and a maximum resolution of 12 Å. Two major classes were selected, and multi-reference classification was performed using heterogeneous refinement with a box size of 128 pixels. The class in which TniQ was bound was selected, and particles re-extracted at 460 pixels were imported into RELION-3.1.3 [50] and then subjected to 3D auto-refine. CTF refinement was performed to adjust beam tilt, anisotropic magnification, and per-particle defocus parameters. Per-particle motion correction was carried out using Bayesian polishing. To further analyze structural heterogeneity, shiny particles were classified by focused 3D classification without alignment (*K* = 3, *T* = 30), in which the focused regions included Cas8/5 HB, two TniQ molecules, Cas7.1, and Cas6. Particles in selected class were restored to the original particles and subjected to 3D auto-refine and 3D classification without alignment (*K* = 4, *T* = 30). The major class was re-imported to CryoSPARC™ v4.2.1 for Non-Uniform Refinement. Resolution was assessed using the 0.143 gold-standard FSC criterion [49]. Local resolution was estimated as described above. Regions including Cas8/5 HB, TniQ, Cas7.1, and Cas6 were further refined by Local Refinement. Focused maps were sharpened using DeepEMhancer [51]. Statistics of cryo-EM data processing are summarized in Table 1.

**Table 1.** Cryo-EM data collection, refinement, and validation statistics

	TniQ-Cascade-dsDNAPR stateEMD-65338PDB 9VTP	TniQ-Cascade-dsDNAFR1 stateEMD-65339PDB 9VTQ	TniQ-Cascade-dsDNAFR2 stateEMD-65340PDB 9VTR
Data collection and processing			
Microscope	CRYOARM^TM^ 300	CRYOARM^TM^ 300	CRYOARM^TM^ 300
Detector	Gatan K3 camera	Gatan K3 camera	Gatan K3 camera
Automation software	SerialEM	SerialEM	SerialEM
Nominal magnification	60000	60000	60000
Acceleration voltage (kV)	300	300	300
Total electron exposure (e^−^/Å^2^)	50.31	49.98	49.92
Defocus range (μm)	−1.4 to −1.6	−1.4 to −1.6	−1.4 to −1.6
Pixel size (Å)	0.752	0.752	0.752
Symmetry imposed	C1	C1	C1
Movies (No.)	4906	8388	7926
Initial particle images (No.)	1095222	1254525	704331
Final particle images (No.)	31509	36247	12291
Map resolution (Å)	2.7	2.8	2.9
FSC threshold	0.143	0.143	0.143
Map sharpening B factor (Å^2^)	−51.9	−53.5	−51.0
3DFSC sphericity	0.670	0.940	0.747
Refinement			
Refinement software	Phenix	Phenix	Phenix
Initial model used	FR2 state	FR2 state	AlphaFold2, 6PIJ
Model resolution (Å)	3.3	3.2	3.4
FSC threshold	0.5	0.5	0.5
Model-map CC (CCmask/CCbox)	0.79/0.76	0.85/0.84	0.82/0.76
Model composition			
Non-hydrogen atoms	29823	29932	29337
Protein residues	3549	3520	3495
Nucleotide residues	98	99	100
Ligands	1	1	1
Average B factors (Å^2^)			
Protein	97.42	110.69	98.59
Nucleotide	90.80	95.64	90.46
Ligand	42.71	55.79	37.98
R.m.s. deviations			
Bond lengths (Å)	0.003	0.003	0.003
Bond angles (°)	0.570	0.544	0.602
Validation			
MolProbity score	2.07	2.03	2.21
Cβ outliers (%)	0	0	0
CaBLAM outliers (%)	2.96	2.45	2.44
Clashscore	6.73	5.88	7.00
Poor rotamers (%)	2.90	2.91	3.87
Ramachandran plot			
Favored (%)	94.99	94.82	94.55
Allowed (%)	5.01	5.18	5.45
Disallowed (%)	0	0	0

### Model building

The model of Cas7 from *V. parahaemolyticus* RIMD 2210633 was generated by homology modeling with SWISS-MODEL [52], employing the Cas7 structure from *V. cholerae* HE-45 (PDB ID: 6PIJ) [28] as the template. Initial models of the other protein components (Cas8/5, Cas6, and TniQ) were generated using AlphaFold2 [53]. These models were rigidly fitted into the cryo-EM maps, using either the Dock in Map function in Phenix [54] or the Fit in Map function in ChimeraX [55]. The models of nucleic acids were manually built using Coot [56]. Subsequent refinement and manual model rebuilding were iteratively performed using real-space refinement in Phenix [54] and Coot, respectively. The final statistics of the refined atomic models are summarized in Table 1. Figures were visualized using ChimeraX [55].

### *In vivo* transposition into the target plasmids

*E. coli* strain BL21(DE3) was initially transformed with the plasmids pETDuet-1-StuI_Donor-*tiaS* and pCOLADuet-1_TnsABC. The former served as the donor plasmid and contained a partial DNA fragment of the *tiaS* gene, which functioned as the cargo DNA in this assay. Competent cells were prepared from the transformants and subsequently transformed with the plasmids pCDFDuet-1_TniQ-Cascade_Target-*tetR* and pACYC184, with the latter serving as the target plasmid. The pCDFDuet-1_TniQ-Cascade_Target-*tetR* plasmid was designed to express TniQ-Cascade programmed to target the tetracycline resistance gene encoded on pACYC184. The cells were re-plated onto LB agar plates supplemented with 50 μg/ml ampicillin, 50 μg/ml kanamycin, 50 μg/ml streptomycin, 34 μg/ml chloramphenicol, and 0.1 mM IPTG, and incubated at 25°C for 48 h. All resulting colonies were collected, and total plasmid DNAs were extracted using a FastGene Plasmid Mini Kit (NIPPON Genetics).

Quantitative PCR (qPCR) was performed to monitor the transposition-derived amplicons under the following conditions: denaturation at 95°C for 5 s, followed by annealing and extension at 60°C for 30 s, repeated for a total of 40 cycles. Each reaction contained 1 × TB Green^®^ Premix Ex Taq™ II (Tli RNaseH Plus) (TaKaRa), 0.2 μM of each primer, and 0.01 ng of total plasmid DNA as the template. The first primer pair was designed to detect both pACYC184 (non-integrated, original plasmid) and pACYC184_Insert (cargo DNA-integrated plasmid). The second primer pair was designed to detect transposed plasmid DNA in which the right end of the CAST was positioned adjacent to the protospacer. The third primer pair was designed to detect the transposition where the left end of the CAST was positioned near the protospacer in the opposite orientation. Following thermal cycling, a melting curve analysis was performed (95°C for 15 s, 60°C for 30 s, and 95°C for 15 s) to confirm the absence of nonspecific amplification or contamination. The *C*_t_ values were determined using the Second Derivative Maximum method. To correct for differences in amplification efficiencies among the three amplicons, standard curves were generated using known concentrations of control plasmids to correlate *C*_t_ values with copy numbers. Transposition efficiency was calculated as (pACYC184_Insert-RL + pACYC184_Insert-LR) / (pACYC184 + pACYC184_Insert-RL + pACYC184_Insert-LR).

To examine transposition into *hflK*, which contains mismatches within the seed region between the TS and the native crRNA, pACYC184_*hflKC* harboring the full-length *hflKC* operon was first used. Transformation, induction, and plasmid extraction were performed as described above. Transposition was detected by PCR. To quantitatively investigate the mismatch tolerance between the crRNA and the TS, the transposition experiment was conducted using a plasmid DNA containing the *hflK* protospacer sequence (pTarget-32). qPCR was performed to determine the transposition efficiency, as described earlier.

### Genome engineering in *E. coli* cells

Methionine auxotroph *E. coli* strain B834(DE3) cells were co-transformed with the plasmids pETDuet-1-StuI_Donor-*metE* and pCOLADuet-1_TnsABC. The transformants were used to prepare competent cells, which were subsequently transformed with pCDFDuet-1_TniQ-Cascade_Target_*metE*ΔC. The resulting transformants were re-plated onto LB agar plates supplemented with 50 μg/ml ampicillin, 50 μg/ml kanamycin, 50 μg/ml streptomycin, and 0.1 mM IPTG and incubated at 25°C for 48 h.

Since pETDuet-1-StuI_Donor-*metE* lacks the T7 promoter upstream of the MCS1, expression of the *metE* gene was not expected to be induced even in the presence of IPTG. However, to rule out the possibility of leaky *metE* gene expression from this plasmid, the cells were further transformed with pHSG398, a plasmid carrying an origin of replication incompatible with that of pETDuet-1-StuI_Donor-*metE*. Because pHSG398 is a high-copy plasmid, the pETDuet-1-StuI_Donor-*metE* plasmid would be excluded from the resulting colonies when the transformants were selected under conditions containing chloramphenicol but lacking ampicillin. To this end, the cells were plated on LB agar plates containing 50 μg/ml kanamycin, 50 μg/ml streptomycin, and 34 μg/ml chloramphenicol. Subsequently, the transformants were cultured and re-plated on agar plates containing Se-Met core medium (FUJIFILM Wako Pure Chemical Corporation), with or without 30 mg/L methionine, supplemented with 50 μg/mL kanamycin, 50 μg/mL streptomycin, and 34 μg/mL chloramphenicol. Transposition was detected by colony PCR. PCR products were analyzed by electrophoresis on a 1.5% agarose gel and by Sanger sequencing to determine the transposition sites.

### Multiple sequence alignment

To compare the types I-F1 and I-F3 CRISPR-Cas systems, bacterial strains harboring both systems were selected based on the previous report [26], and their amino acid sequences were extracted. Furthermore, sequences from structurally characterized proteins were deliberately included to facilitate structural comparisons. The collected sequences were clustered using CD-HIT [57] with a sequence identity threshold of 95%. These clustered sequences were then used as input for Clustal Omega [58] to produce a multiple sequence alignment of the amino acid sequences.

## Results

### Cryo-EM structures of *Vibrio parahaemolyticus* type I-F3 TniQ-cascade

**Figure 1.**
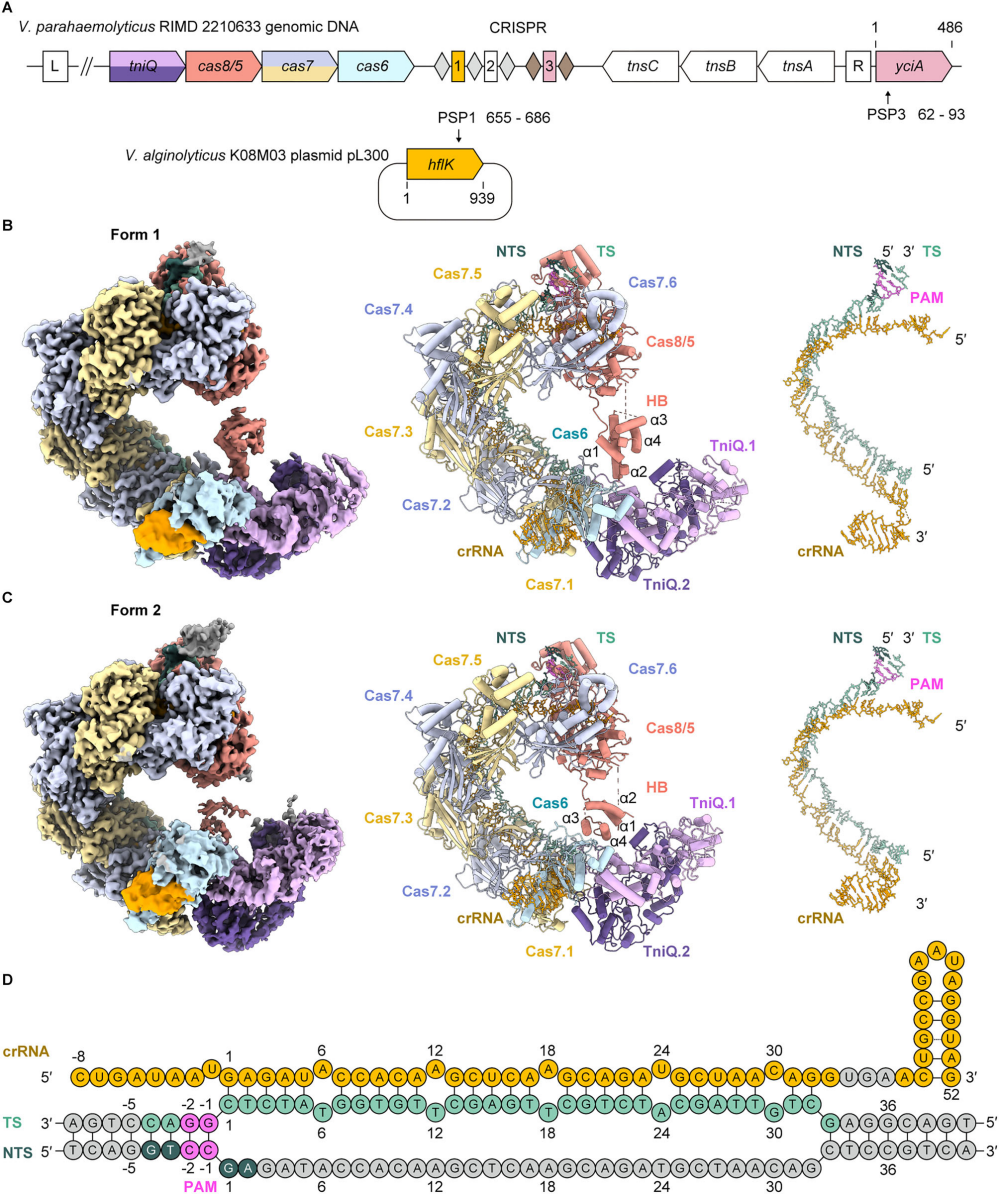
Overall structure of type I-F3 TniQ-Cascade from *V. parahaemolyticus*. (**A**) Schematic diagram of the CAST in *V. parahaemolyticus* RIMD 2210633 (top) and the *hflK* gene on *V. alginolyticus* K08M03 plasmid pL300 (bottom). Nucleotide numberings for the *yciA* and *hflK* genes, as well as the protospacers (PSP) targeted by the first and the third crRNAs, are indicated. (**B, C**) Overall structures of target DNA-bound VpTniQ-Cascade in form 1 and form 2, respectively. (Left) Cryo-EM map of the complex. (Middle) Cartoon representation of the complex. The numbering of α-helices in the Cas8/5 HB is indicated. (Right) Stick representations of the target DNA and crRNA within the complex. (**D**) Schematic diagram of the R-loop structure between the crRNA derived from the first spacer of the CRISPR array in panel (A) and its perfectly matched target DNA. The crRNA, TS, and NTS are colored yellow, light green, and dark green, respectively. The PAM sequence is pink. Disordered nucleotides in the form 2 structure are colored gray. Nucleotide numberings of each strand are indicated.

We identified the genes encoding Cas proteins, crRNAs, and transposition-associated proteins that mediate RNA-guided DNA transposition in chromosome 2 of *V. parahaemolyticus* RIMD 2210633 (Fig. 1A). The CRISPR-Cas effector components of VpCAST are classified as the type I-F3a system. As reported previously, the spacer sequence between atypical repeats is homologous to the 5′-terminal region of the *yciA* gene (Fig. 1A), which is located adjacent to VpCAST and serves as the attachment (*att*) site for transposition [26]. Additionally, we searched for probable target sequences of the typical crRNAs, using Nucleotide BLAST. The first of the two typical crRNAs exhibits a near-perfect match (90.6%) to the *hflK* gene on the plasmid carried by *Vibrio alginolyticus* (Fig. 1A). The target sequences of the second typical crRNA could not be identified in the database. Although the CAST system shows evidence of site-specific mobility between plasmid and genomic DNA, further research is needed to clarify how TniQ-Cascade specifically recognizes its target DNA. To address this, we sought to elucidate how VpTniQ-Cascade binds to its target DNA and directs it to the transposition machinery. We first reconstituted the transposition reaction of VpCAST in *E. coli* strain BL21(DE3) to assess its functionality (Supplementary Fig. S4A). Bidirectional transposition into the *lacZ* gene was detected by PCR (Supplementary Fig. S4B). Sanger sequencing analysis revealed that transposition occurred 49 or 51 bp away from the protospacer, which is generally consistent with previous reports on type I-F3 CAST systems from other species (Supplementary Fig. S4C and D) (21,39). VpTniQ-Cascade was subsequently purified and incubated with dsDNA to reconstitute the target DNA-bound form. The resulting complex containing all protein components, the crRNA, and the target DNA was further purified (Supplementary Fig. S5).

The structure of the target DNA-bound VpTniQ-Cascade was determined by cryo-EM single-particle analysis in two distinct states, designated as form 1 and form 2, at 2.8 and 2.9 Å resolutions, respectively (Fig. 1B–D, Supplementary Figs S1 and  S2, and Table 1). Like the previously reported structures [27–29, 43, 44], TniQ-Cascade exhibits a helical architecture, in which one Cas8/5 molecule and two TniQ molecules (TniQ.1 and TniQ.2) are located at the two ends of the complex, and six Cas7 subunits (Cas7.1–Cas7.6) constitute its helical backbone. Cas6 is bound to the stem-loop of the 3′ region of the crRNA. Cas8/5 has a flexible HB domain, which is arranged to connect both ends of the helical complex structure (Fig. 1B and C). The relative location of the Cas8/5 HB domain in the complex is different between form 1 and form 2. In form 1, α-helices 1 and 2 of the HB domain are in the proximity of TniQ.1, whereas in form 2, they are separated from it (Fig. 1B and C). This study indicates the importance of the structural difference between these two states in suitably positioning the target DNA for the subsequent transposition reaction (see below). Given the higher structural quality of form 1, we primarily describe the features of the form 1 structure unless otherwise specified.

### PAM recognition and promiscuous PAM preference by TniQ-Cascade

**Figure 2.**
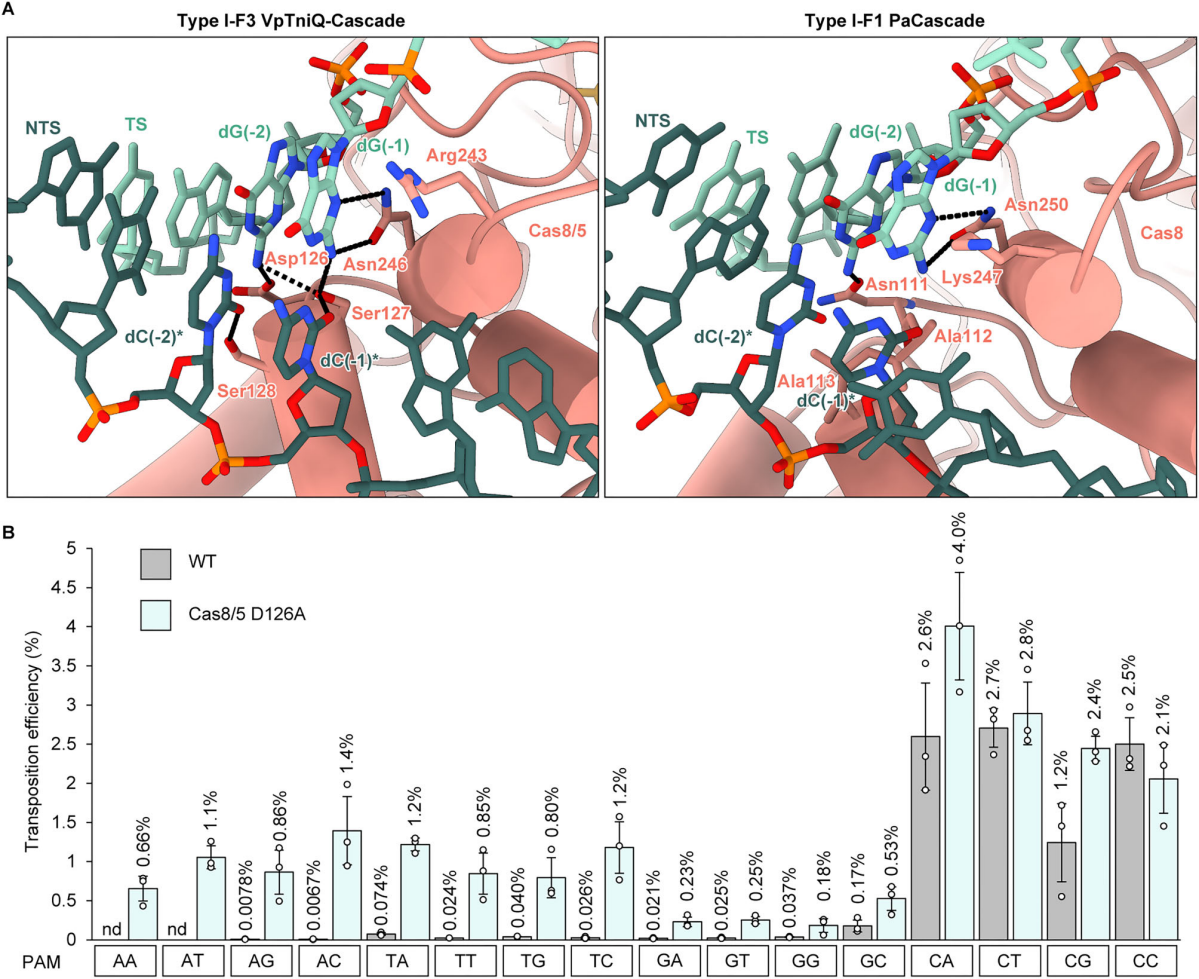
PAM recognition mechanism by Cas8/5 of VpTniQ-Cascade. (**A**) Structural comparison of the PAM recognition mechanism between the type I-F3 VpTniQ-Cascade (left) and the type I-F1 PaCascade (right; PDB ID: 6NE0). Cas8/5 of the VpTniQ-Cascade and Cas8 of the PaCascade are shown as cartoon representations. The TS and NTS are depicted as light green and dark green stick models, respectively. Dashed lines indicate hydrogen bonds. (**B**) *In vivo* transposition assay of the wild-type and Cas8/5 D126A mutant-containing VpTniQ-Cascade for 16 different PAM sequences. Transposition efficiencies are plotted. Data are presented as mean ± SD from three independent experiments. The mean transposition efficiency is indicated. nd, not detected.

The cryo-EM structures of the VpTniQ-Cascade reveal a unique PAM recognition mechanism mediated by Cas8/5, which differs from that employed by the type I-F1 Cascade. The PAM is enclosed by a region of Cas8/5 comprising two α-helices (residues 126–135 and 242–247) and a loop (residues 459–469). Arg243 disrupts the base pairing at the PAM-proximal end of the protospacer by acting as a wedge, in a manner similar to that observed in previously reported structures of the type I Cascade (Fig. 2A and Supplementary Fig. S6) [11, 13, 15, 16, 18, 28, 32, 59]. The side chains of Ser127 and Ser128 form hydrogen bonds with the nucleobases of dC(−1)* and dC(−2)* in the PAM, respectively, on the minor groove side of the non-target strand (NTS; asterisk indicates nucleotide on the NTS). Regarding the complementary nucleotides of the 5′-CC-3′ PAM, the exocyclic amino group of dG(-2) of the target strand (TS) is within hydrogen bonding distance of the side chains of Asp126 and Ser127, while dG(−1) forms bidentate hydrogen bonds with the side chain of Asn246 (Fig. 2A). The N2 atom of dG(−1) further forms a hydrogen bond with the side chain of Ser127. Among these interactive residues, Asp126 of VpCas8/5 is conserved as either aspartate or asparagine in both type I-F1 and type I-F3 Cascades (Supplementary Fig. S7A). In contrast, Ser127 of VpCas8/5 is strictly conserved in the type I-F3 CAST system, while the corresponding residue in the type I-F1 adaptive immune system is alanine (Supplementary Fig. S7A). In the cryo-EM structures of the *V. cholerae* and *A. salmonicida* TniQ-Cascades bound to target DNA [27–29, 43, 44], these residues occupy positions similar to the corresponding residues in that of *V. parahaemolyticus*, suggesting that Cas8/5 in the TniQ-Cascade recognizes the PAM in a similar manner across other species. Previous cryo-EM studies of the *V. cholerae* and *A. salmonicida* TniQ-Cascades have greatly advanced our understanding of their complex architectures [27, 28, 29, 43, 44]. However, due to limitations in the achievable resolution, some details of PAM recognition could not be fully visualized. Our study addresses this gap by providing a more detailed view of the interactions critical for PAM recognition in TniQ-Cascade.

To explore the roles of these residues in PAM recognition during the transposition reaction, we first investigated the PAM preference of VpTniQ-Cascade by evaluating the transposition efficiencies of target plasmids encoding all 16 possible combinations of dinucleotides at positions −2 and −1 of the PAM. The transposition experiment with wild-type VpTniQ-Cascade clearly demonstrated a preference for dC(−2)*, whereas no nucleotide preference was observed at position −1 (Fig. 2B). This result shows that VpTniQ-Cascade recognizes a 5′-CN-3′ PAM, which is consistent with the PAM preferences previously reported for those from *V. cholerae* and *A. salmonicida* [21, 38, 39]. The residue corresponding to Asp126, which forms a hydrogen bond with the N2 atom of dG(−2), is widely conserved as either aspartic acid or asparagine among the type I-F CRISPR-Cas systems in both CAST and adaptive immunity systems (Supplementary Fig. S7A). In contrast to the wild-type, the D126A mutant exhibited transposition activity not only with the PAMs containing dC(−2)*, but also with those containing the three other nucleobases (Fig. 2B). Surprisingly, the preferences for the PAMs containing dA(−2)* and dT(−2)* increased by ~100–200 fold and 16–45 fold, respectively, compared to the wild-type (Fig. 2B). These findings indicate that Asp126 restricts the accommodation of alternative nucleotides and acts as a key determinant of the PAM preference at position −2 of VpTniQ-Cascade.

Ser127 is well conserved among Cas8/5 proteins, whereas the residue at position 128 is more divergent (Supplementary Fig. S7A). Interestingly, in the type I-F1 Cascade, the corresponding residues in Cas8 are both substituted with alanine [18], indicating that no hydrogen bonds are formed with the PAM. Given the high conservation of these alanine residues in Cas8 of the type I-F1 Cascade (Supplementary Fig. S7A), we hypothesized that their presence at these positions contributes to the preference of cytidine at position −1 of the PAM. However, contrary to our expectations, individual single mutations as well as double mutations retained transposition efficiencies comparable to those of wild-type TniQ-Cascade, without exhibiting any nucleotide preference at position −1 (Supplementary Fig. S7B). Accordingly, in the type I-F3 TniQ-Cascade, the presence of serine residue(s) does not contribute to the PAM preference at position −1, despite the residue's ability to form hydrogen bonds with the PAM nucleotides.

### DNA transposition with mismatched pairs between crRNA–TS heteroduplex

The first typical spacer of the CRISPR array in VpCAST almost perfectly matches the *hflK* gene (nucleotides 655–686) in *V. alginolyticus* K08M03 plasmid pL300, which contains a 5′-CC-3′ motif as the PAM (Fig. 1A and Supplementary Fig. S8). The resulting crRNA–TS heteroduplex contains 3 bp mismatches, with the first and second nucleotides of the crRNA guide sequence failing to base pair with the TS at the PAM-proximal site. In contrast, a mismatched pair between A(12) of the crRNA and dC(12) of the TS is located at one of the flipped-out positions that occurs at every sixth nucleotide interval, which is commonly observed in the class 1 CRISPR-Cas effectors [11, 13, 15, 16, 18, 59–61]. Therefore, the mismatch at position 12 does not affect Cascade binding to the target DNA. Interestingly, the first and second base pairs constitute the seed sequence in the type I CRISPR-Cas system [42, 62–64]. Base pairing within the seed sequence is known to be crucial for target DNA binding and subsequent degradation in the CRISPR-Cas systems responsible for adaptive immunity [40, 42, 65–67]. Accordingly, it remained unclear whether type I-F3 CAST systems are capable of integrating the transposon into candidate target genes bearing a few TS-crRNA guide mismatches in the seed sequence.

**Figure 3.**
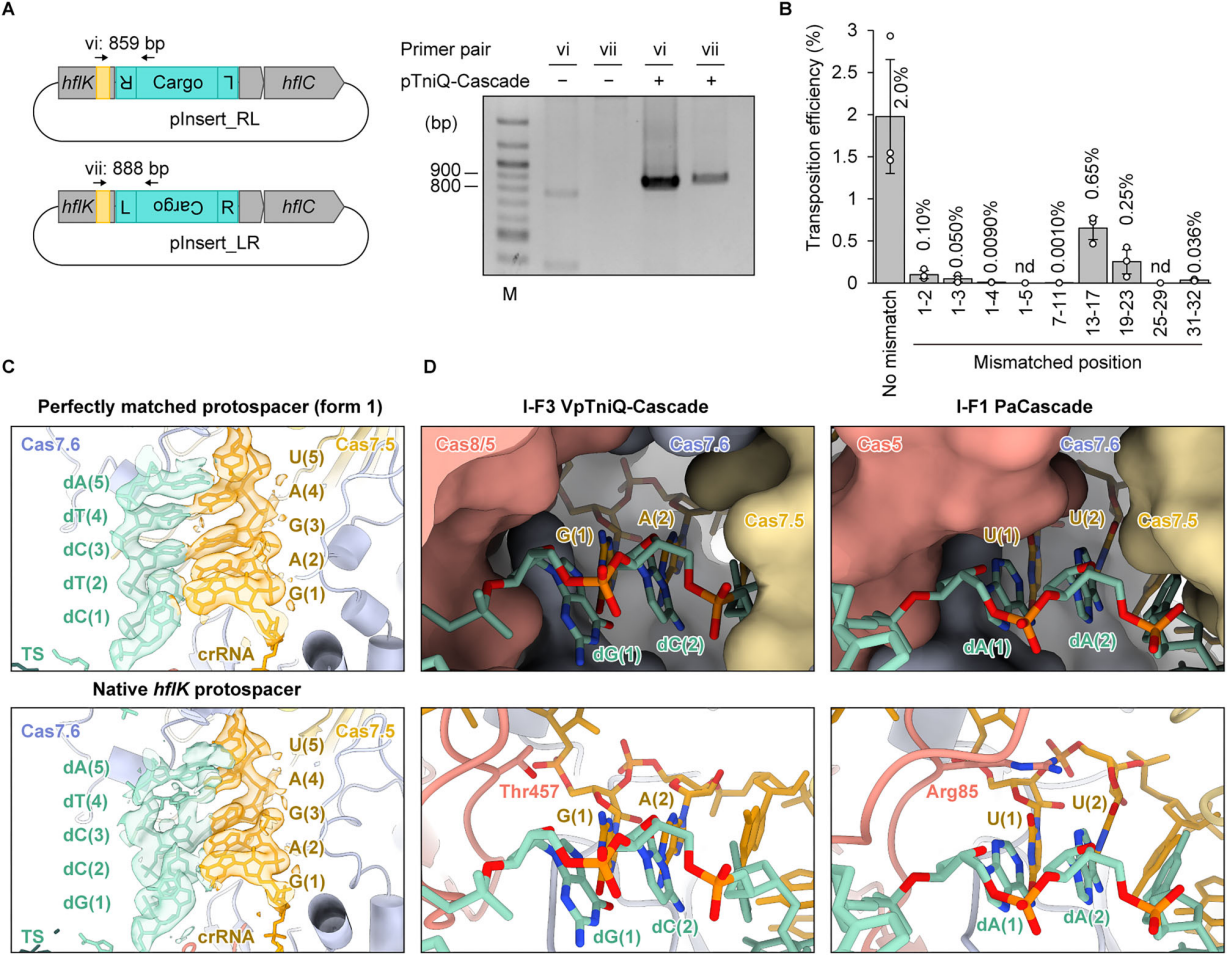
Effects of mismatched base pairs on transposition. (**A**) *In vivo* reconstitution of transposition into the native protospacer of the *hflK* gene. The schematic diagram depicts transposition into a target plasmid containing the *hflK–hflC* gene operon, resulting in transposed pInsert plasmids in both forward and reverse orientations (left). The positions of primer pairs and the sizes of PCR amplicons are indicated above the corresponding regions. The amplicons were analyzed by agarose gel electrophoresis (right). The gel image is representative of three independent experiments. (**B**) *In vivo* transposition efficiencies with crRNA and mismatched target DNA. Transposition efficiencies, evaluated by qPCR, are plotted. Data are presented as mean ± SD from three independent experiments. The mean transposition efficiency is indicated above each bar. nd, not detected. (**C**) Structural comparison of the seed region between the perfectly matched protospacer (top; form 1) and the *hflK* protospacer (bottom). The crRNA and the TS are depicted as stick representations. Cryo-EM maps for the first five base pairs of the heteroduplex in each structure are shown.(**D**) Structural comparison of the seed regions between the VpTniQ-Cascade containing mismatched base pairs (left) and PaCascade (right). Protein subunits are shown as surface models (top) and cartoon models (bottom). The Thr457 residue of Cas8/5 in VpTniQ-Cascade and its corresponding residue, Arg85 of Cas8 in PaCascade, are depicted as stick representations.

To investigate this possibility, we initially tested whether a DNA fragment containing the left and right ends of VpCAST could be inserted into the *hflK* gene, despite the presence of mismatches in the seed region when hybridized with the guide sequence of the crRNA. A plasmid carrying full-length *hflK* and its downstream *hflC* genes was used as the target for transposition. Notably, the DNA integration occurred downstream of the predicted protospacer sequence, as confirmed by PCR (Fig. 3A). To quantitatively assess the transposition efficiency, we constructed a target plasmid (pTarget-32) containing a 32 bp protospacer derived from the *hflK* gene. Although the transposition efficiency for this mismatched target was reduced 20-fold compared to that with a perfectly matched target DNA, VpTniQ-Cascade and transposition-associated proteins successfully catalyzed transposition *in vivo* (Fig. 3B and Supplementary Fig. S9A). To determine the integration sites of the cargo DNA, we conducted Sanger sequencing of the resulting PCR products. The electropherogram obtained from the mismatched target sample revealed that the cargo DNA was predominantly integrated 49 bp downstream of the protospacer sequence, the same site observed with the perfectly matched DNA (Supplementary Fig. S9B).

It is conceivable that the reduced transposition efficiency at the *hflK* gene reflects the use of a CG motif at NTS positions 1 and 2 as a suboptimal PAM, which may direct TniQ-Cascade to recognize a 30 nt protospacer (Fig. 2B and Supplementary Fig. S8A). To test this possibility, we first introduced the optimal CC motif as a possible PAM at NTS positions 1 and 2 of the protospacer (Supplementary Fig. S10A; schematic diagram labeled with 1–2 CC) and assessed whether this would enhance the transposition efficiency. The resulting construct did not increase the transposition efficiency (Supplementary Fig. S10B). Next, we employed the Cas8/5 D126A mutant, which preferentially recognizes a CG PAM (Fig. 2B), to further evaluate this hypothesis. For a target DNA bearing a CG motif at NTS positions 1 and 2 (Supplementary Fig. S10A; schematic diagram labeled with 1–2 CG), no increase in transposition efficiency was observed relative to the wild-type (Supplementary Fig. S10B). Collectively, these results suggest that VpTniQ-Cascade recognizes the CC PAM when targeting the *hflK* gene, thereby ruling out the possibility that the reduced transposition efficiency arises from the recognition of the CG motif as a suboptimal PAM; instead, it is more likely attributable to mismatched base pairs between the crRNA and the TS. Although transposition for targeting the *hflK* gene is less efficient, the fact that DNA integration into the target with several mismatches at the PAM-proximal region occurs is highly significant from the perspective of the functional relevance of the CAST system (see “Discussion” section).

To understand how the partially mismatched heteroduplex is bound in the complex, we determined the cryo-EM structure of VpTniQ-Cascade bound to a target DNA containing the *hflK* protospacer at a resolution of 2.7 Å (Fig. 3C, Supplementary Figs S3 and  S8, and Table 1). While the overall architecture of VpTniQ-Cascade closely resembles those of form 1 and form 2 (Fig. 1 and Supplementary Fig. S8), the crRNA–TS base pairing at the PAM-proximal site is distinct. Unlike the canonical Watson–Crick base pairing observed in form 1 and form 2, the cryo-EM maps of VpTniQ-Cascade containing the *hflK* protospacer clearly reveal the formation of a Hoogsteen G–G base pair and a mismatched A–C base pair at the first and second positions of the seed region, respectively, which nonetheless fit snugly within the heteroduplex-binding channel (Fig. 3C). Unexpectedly, although the cryo-EM maps for the first and second positions are well defined, the map at the fourth position is weaker, suggesting local destabilization and/or conformational flexibility at this site in the heteroduplex (Fig. 3C). These findings indicate that in the type I-F3 CAST system, mismatched base pairs at the first two positions are partially tolerated even at the PAM-proximal site. However, such mismatches may introduce structural strain that impairs base pairing in downstream regions.

To clarify the mismatch tolerance at the PAM-proximal site, we further examined the structural differences around the seed region between the type I-F3 VpTniQ-Cascade and the type I-F1 *Pseudomonas aeruginosa* (Pa) Cascade [18]. In the structure of PaCascade, Arg85 of Cas5 occupies the space above the first and second base pairs in the seed region (Fig. 3D), thereby stabilizing the heteroduplex and potentially preventing the formation of fragile mismatched base pairs at these positions. In contrast, the corresponding residue in Cas8/5 of VpTniQ-Cascade is Thr457, whose side chain is too short to interact with the heteroduplex as a lid. Consequently, no residue contacts the first and second seed positions in the VpTniQ-Cascade structure (Fig. 3D), suggesting the presence of space that may accommodate flexible mismatched pairs at these positions. Similar amino acid substitutions are observed in the Cas8/5 homologs of the type I-F3 CAST systems from other species (Supplementary Fig. S11) [27, 28]. Collectively, these structural features near the seed region may mitigate the energetically unfavorable binding pose of TniQ-Cascade caused by mismatches.

To further investigate the mismatch tolerance in other regions of the heteroduplex, we constructed a series of target plasmids based on pTarget-32, which generated various mismatches between the crRNA and TS, and examined the transposition efficiencies by qPCR. As the mismatches accumulated within the first 6-nt segment, the transposition efficiency progressively declined, and complete mismatches in this segment abolished the transposition activity (Fig. 3B and Supplementary Fig. S9A). This result suggests that several mismatches at the PAM-proximal site can be tolerated, but accurate base pairing is crucial for the efficiency of the subsequent reaction, similar to the behavior of the type I-F1 Cascade [67]. The introduction of mismatches into the second segment (mismatched positions 7–11) also severely impaired transposition, indicating that the first two 6-nt segments act as the functional seed sequence of TniQ-Cascade. In contrast, mismatches introduced in the third (mismatched positions 13–17) and fourth (mismatched positions 19–23) segments had minimal effects on the transposition efficiency. However, mismatches in the fifth segment (mismatched positions 25–29) completely abolished transposition (Fig. 3B and Supplementary Fig. S9A), consistent with previous reports on the CAST from *V. cholerae* [21, 36]. Interestingly, mismatches at the last two nucleotides of the protospacer also reduced the transposition efficiency by 56-fold (Fig. 3B and Supplementary Fig. S9A). These findings clearly demonstrate that the base pairing at the PAM-distal site is critical for the functionality of TniQ-Cascade, in contrast to the type I-F1 Cascade, which only monitors correct base pairing in the PAM-proximal seed region [41, 67].

### DNA bending at PAM-distal site by interactions between Cas8/5 and TniQ

**Figure 4.**
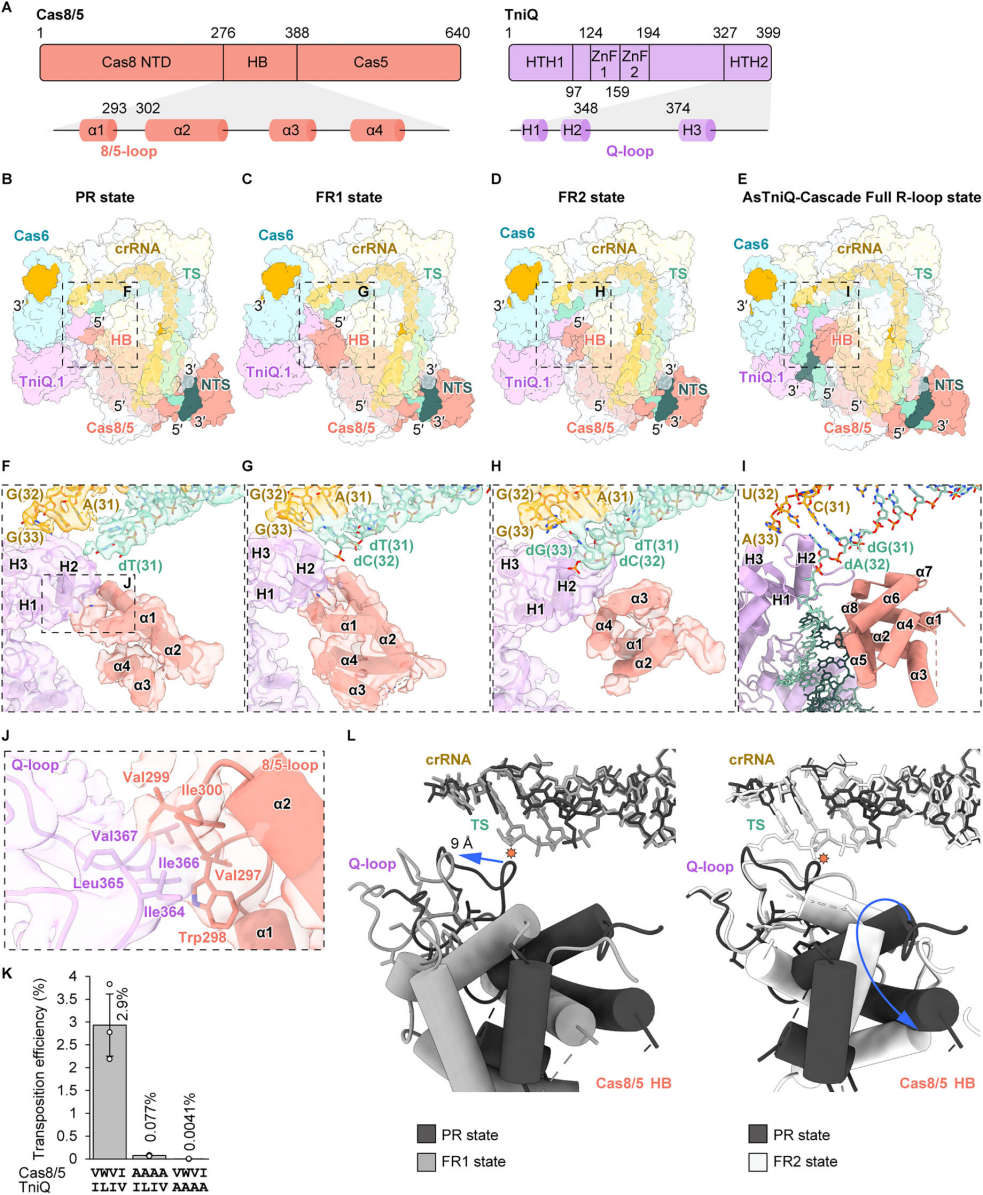
Conformational changes of the Cas8/5 HB and TniQ in coordinating dsDNA accommodation at the PAM-distal region. (**A**) Domain architectures of Cas8/5 and TniQ (top). The secondary structures of the HB domain in Cas8/5 and the HTH2 domain in TniQ are highlighted (bottom). Amino acid residue numbering is indicated. (**B**–**E)** Overall structures of target DNA-bound TniQ-Cascade. Protein subunits and nucleic acids are shown as surface representations, with the six Cas7 subunits and TniQ.2 rendered transparent. Panels (B–E) correspond to the PR state, FR1 state, and FR2 state of VpTniQ-Cascade (this study) and the full R-loop state of AsTniQ-Cascade (PDB ID: 7U5D), respectively. (**F**–**I**) Close-up views of the structures at the PAM-distal site, indicated by dotted lines in panels (B–E), respectively. Panels (F–I) correspond to the PR state, FR1 state, and FR2 state of VpTniQ-Cascade, and the full R-loop state of AsTniQ-Cascade, respectively. The α-helices of the Cas8/5 HB and TniQ.1 HTH2 are labeled. The model is shown together with the cryo-EM focused map in panels F–H. (**J**) Detailed view of the interactions between the 8/5-loop and the Q-loop in the PR state, as indicated by dotted lines in panel (F). The atomic model is shown together with the cryo-EM focused map. Hydrophobic residues at the interface are shown in stick models. (**K**) *In vivo* transposition assay of wild-type VpTniQ-Cascade and its variants containing quadruple mutations in the 8/5-loop or Q-loop. Transposition efficiencies are shown as bar plots. Data are presented as mean ± SD from three independent experiments. The mean transposition efficiency is indicated above each bar. **(L)** Structural comparisons of the interface involving the Cas8/5 HB, the Q-loop, and the heteroduplex at the PAM-distal site between the PR and FR1 states (left) and between the PR and FR2 states (right). For these comparisons, the TniQ-Cascade structures were superimposed using Cas7.1 as the reference. Orange octagrams indicate steric clashes between the Q-loop in the PR state and the TS in the FR1 state (left), as well as between the Q-loop in the PR state and the TS in the FR2 state (right).

Mismatches at positions 25–29 and 31–32 abolished and markedly reduced the transposition activity of VpCAST, respectively, suggesting that correct base pairings between the guide sequence of the crRNA and the TS at the PAM-distal site are crucial for DNA transposition (Fig. 3B). To better understand how the PAM-distal site conformation affects transposition, we compared this region among the three cryo-EM structures (Fig. 4). The complex bound to the native *hflK* protospacer adopts a partial R-loop (PR) state, where the first 29 nt of the TS (corresponding to positions 1–29 in the 3′-5′ direction) form a heteroduplex with the crRNA guide, while the last two nucleotides remain unpaired (Fig. 4B and F). In contrast, the form 1 and form 2 TniQ-Cascades adopt full R-loop states, FR1 and FR2, respectively, with complete base pairings across the entire protospacer (Fig. 4C, D, G, and H).

During the single-particle analysis, we noticed that the orientations of the Cas8/5 HB relative to the rest of the VpTniQ-Cascade differ among the three structures (Fig. 1B and C; Supplementary Fig. S8). However, due to the intrinsic flexibility of this region, its precise conformation was ambiguous, particularly in the FR2 and PR states. To visualize the Cas8/5 HB in detail across these three states, we calculated focused maps through Local Refinement after several rounds of 3D classification without alignment (Supplementary Figs S1–S3). As a result, although the local resolution is lower than that of other regions, we successfully obtained maps of sufficient quality to trace the α-helices within the Cas8/5 HB domain in all three states (Fig. 4F–H and Supplementary Figs S1K, S2L, and S3M). Structural comparisons of the resulting models of the three distinct conformational states revealed different interaction modes between the Cas8/5 HB domain and the second helix–turn–helix (HTH2) domain of TniQ.1 (Fig. 4A and F–H). The Cas8/5 HB and TniQ HTH2 domains each possess an idiosyncratic loop enriched in hydrophobic residues: the loop between helices α1 and α2 in Cas8/5 HB (8/5-loop) and that between helices H2 and H3 in TniQ HTH2 (Q-loop), respectively.

In the PR state, the Cas8/5 HB and TniQ.1 HTH2 domains form a hydrophobic interface, stabilized by Val297, Trp298, Val299, and Ile300 of the 8/5-loop and Ile364, Leu365, Ile366, and Val367 of the Q-loop (Fig. 4F and J). This hydrophobic interface places the Q-loop of TniQ.1 in a position that sterically clashes with the TS, which prevents base pairing with the crRNA guide at position 31 and causes significant bending of the TS (Fig. 4F). The DNA bending caused by this interaction is presumed to direct the DNA segment in the PAM-distal region toward the HTH1 and ZnF domains of TniQ.1. To assess the functional significance of the hydrophobic interface, quadruple alanine substitutions were introduced into each set of hydrophobic residues, and their transposition activities were evaluated by qPCR. Both mutants exhibited marked reductions in transposition efficiency (Fig. 4K), suggesting that these hydrophobic residues are essential for proper DNA positioning and/or for stabilizing the conformation of the TniQ-Cascade required for subsequent transposition. Despite the disordered PAM-distal DNA in the PR state, it might still be directed toward the ZnF domains of TniQ.1. This bending likely facilitates the dsDNA-binding to TniQ.1, as suggested by the previous study [27].

While the FR1 and FR2 states form similar full R-loop structures, the latter contains an additional single-stranded nucleotide of the TS adjacent to the heteroduplex (Fig. 4H). Furthermore, the relative orientation of the Cas8/5 HB domain differs between the two states (Fig. 4G and H). In the FR1 state, the Cas8/5 HB domain adopts an orientation nearly identical to that observed in the PR state (Fig. 4F and G), thereby retaining the hydrophobic interactions between the 8/5-loop and the Q-loop found in the PR state. Interestingly, to avoid the steric clashes between the base pairing at the PAM-distal site and the hydrophobic interface, the HB domain and two TniQ subunits undergo rigid-body rotation of ~6°, using the domain boundary between the Cas8/5 NTD and HB as a pivot. This conformational rearrangement displaces the 8/5-loop and the Q-loop by ~9 Å, allowing the accommodation of base pairing at the PAM-distal site in TniQ-Cascade (left panel of Fig. 4L).

Comparisons of the three structures revealed that the Cas8/5 HB domain in the FR2 state is rotated (Fig. 4F–H), leading to the loss of the hydrophobic interactions between the 8/5-loop and the Q-loop observed in the former two states. This structural rearrangement altered the conformation of the Q-loop of TniQ.1, enabling it to function as a platform that snugly fits both the TS-crRNA base pairs at the PAM-distal site and the adjacent single-stranded region of the TS (right panel of Fig. 4L). Another prominent feature of the FR2 state is that the two ends of TniQ-Cascade are positioned more closely compared to the PR state, with Cas7.3 acting as a pivot to compact the complex. The hinge-like motion of the two ends of the complex is also observed between the structures of the FR1 and FR2 states.

Given that both the crRNA–TS heteroduplex at the PAM-distal site and the 8/5- and Q-loop-mediated interactions are required for transposition, we asked whether extending the complementarity between the TS and the 3′ repeat-derived region of the crRNA would affect the conformation of the PAM-distal site and thereby influence the transposition efficiency, regardless of the 8/5- and Q-loop-mediated interactions. To this end, we introduced nucleotide substitutions into the target DNA to enable base pairing with crRNA positions 33–37 (Supplementary Fig. S12A; schematic diagram labeled with 33–37 matched). In assays reconstituted with wild-type, 8/5-loop mutant, or Q-loop mutant TniQ-Cascade, these substitutions had no effect on the transposition efficiency under any tested conditions (Supplementary Fig. S12B).

### Functional gene insertion using VpCAST

**Figure 5.**
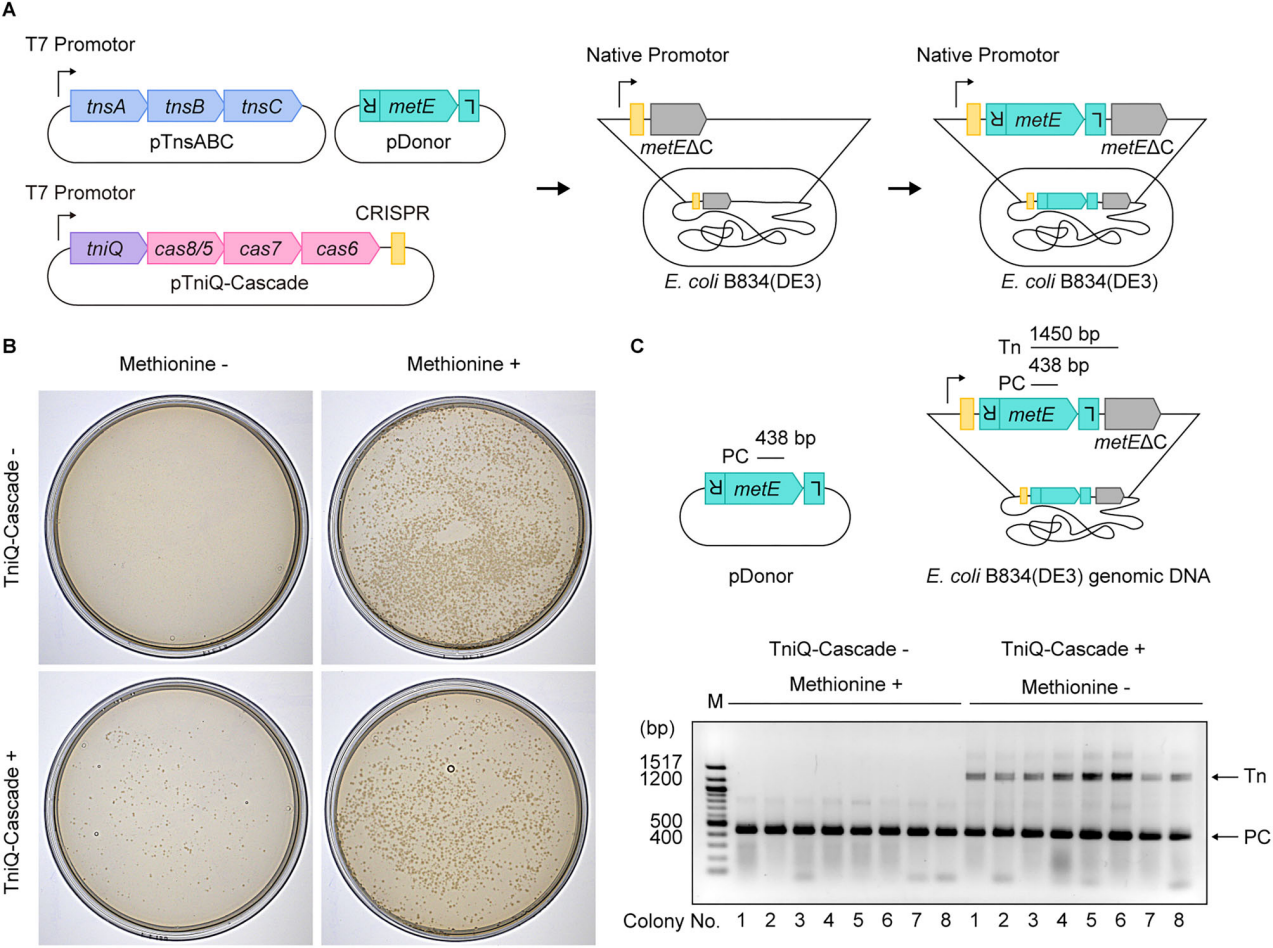
Genome engineering in methionine auxotroph *E. coli* strain B834(DE3). (**A**) Schematic diagram of the *in vivo* methionine synthesis gene complementation experiment. The CRISPR array containing a single spacer targeting the sequence of the native promoter of the *metE* gene on pTniQ-Cascade and the corresponding target sequence on the genomic DNA of *E. coli* strain B834(DE3) are shown in light yellow. (**B**) Growth of each transformant on minimal medium in the presence or absence of methionine. The images of transformants are representative of three independent experiments. (**C**) Detection of TniQ-Cascade-dependent transposition. The DNA regions amplified by colony PCR and their corresponding product sizes are shown (top). The amplicons were analyzed by agarose gel electrophoresis (bottom). Tn and PC denote the fragment amplified from the transposed DNA into the genome and the positive control DNA fragment for PCR, respectively. The gel images are representative of three independent experiments.

We evaluated the utility of the VpCAST system as a site-specific genome editor and assessed the *in vivo* functionality of the genes integrated into the genome. To this end, we used the methionine auxotroph *E. coli* strain B834(DE3) [68], which lacks nucleotides 763–1540 of *metE*, a vitamin B_12_-independent methionine synthesis gene [69]. Due to the absence of the active form of MetE in B834(DE3), the strain is unable to grow under methionine-depleted conditions. Thus, we hypothesized that the incorporation of the intact *metE* gene in the genomic DNA would allow the cells to grow under methionine-depleted conditions (Fig. 5A). The crRNA was designed to target the sequence just downstream of the native promoter of *metE*, which bears a 5′-CT-3′ sequence as a suitable PAM for the transposition reaction (Fig. 2B). As a positive control, colony formation was confirmed on methionine-supplemented medium following transformation with either pTniQ-Cascade or an empty plasmid (Fig. 5B). When grown on minimal medium lacking methionine, colonies were formed by cells transformed with pTniQ-Cascade, but not with an empty plasmid, suggesting that the intact *metE* gene was integrated by the VpCAST system into the genome and expressed adequately. To detect DNA integration into the genome, DNA fragments were amplified by PCR using a pair of primers. The forward primer was designed to anneal to the nucleotide sequence at positions 1277–1299 (i) of *metE*, which lies within the region deleted in B834(DE3). The reverse primer was designed to anneal perfectly to the nucleotide sequence at positions 95–118 (ii) and partially to the sequence at positions 1691–1714 (iii) of *metE* (Fig. 5C and Supplementary Fig. S13). Accordingly, successful genomic transposition would result in the generation of both 1.45 kbp and 438 bp PCR products. Eight colonies were randomly selected from the specimen and the negative control, each of which had been transformed with pTniQ-Cascade or an empty plasmid and cultured on methionine-depleted or methionine-supplemented minimal medium, respectively (Fig. 5C). In contrast to the negative control, all transformants with pTniQ-Cascade produced two bands, with the upper band of 1.45 kbp derived from both the integrated DNA and the endogenous *metE* gene in B834(DE3). The integrity of the integrated DNA sequence was confirmed by Sanger sequencing (Supplementary Fig. S13).

## Discussion

**Figure 6.**
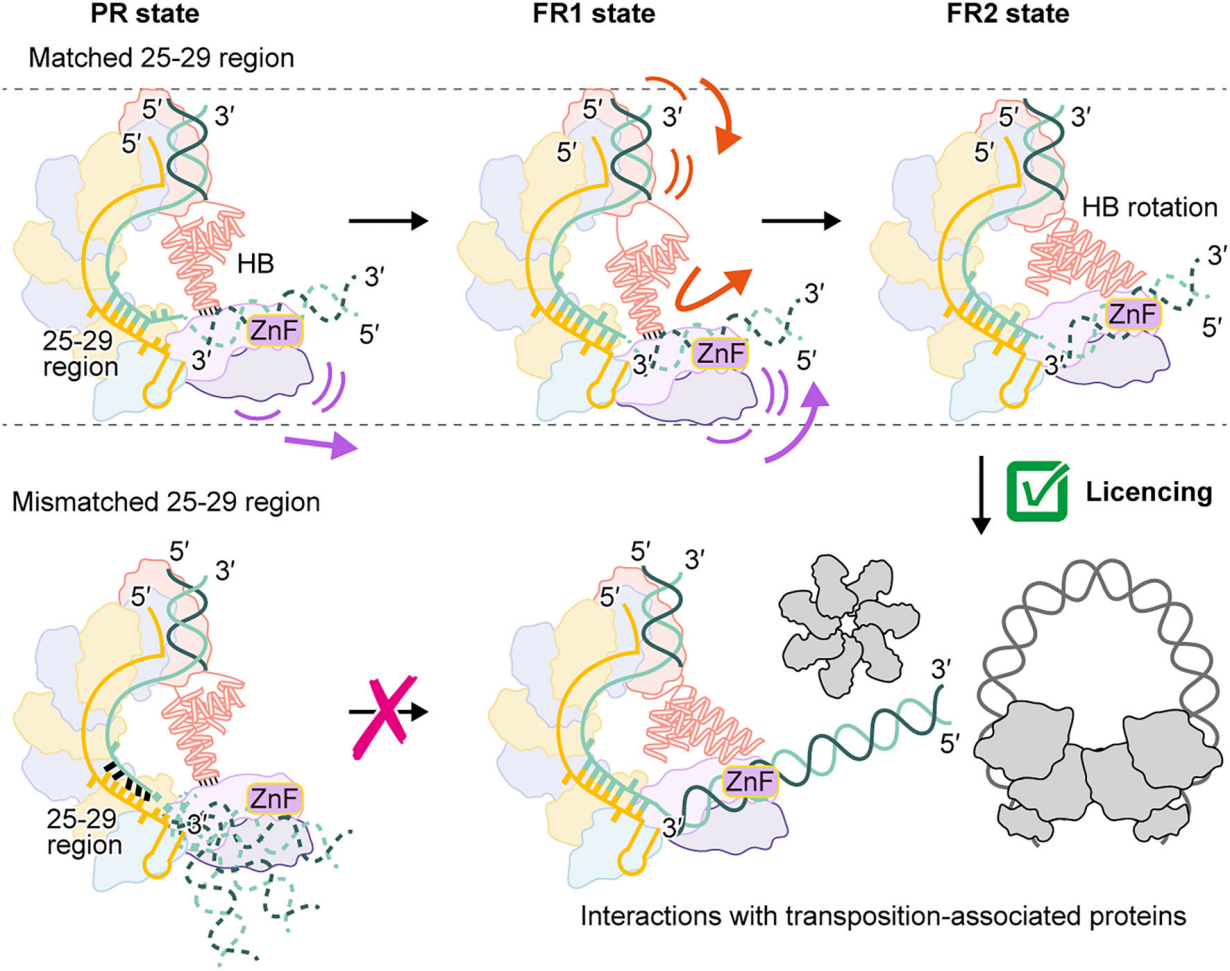
Model of transposition licensing via conformational changes at the PAM-distal site. In the PR state, accurate base pairing at positions 25–29 is monitored, which facilitates the following DNA bending through interactions with the Cas8/5 HB and TniQ.1 (top left). If mismatches are present in the PAM-distal region (mismatched positions 25–29), then proper DNA bending would likely not occur, and as a result, the transposition reaction does not take place (bottom left). The Cas8/5 HB and the two TniQ molecules undergo rigid-body rearrangements to enlarge the space at the TS binding site, enabling the formation of a full R-loop (the FR1 state; top middle). Once the TS reaches TniQ.1, the hydrophobic interactions between the Cas8/5 HB and TniQ.1 are rearranged, resulting in the rotation of the Cas8/5 HB and bringing both ends of TniQ-Cascade closer together (the FR2 state; top right). This structural rearrangement is proposed to stabilize the downstream dsDNA for subsequent reactions. Through these licensing processes, interactions with transposition-associated proteins would be facilitated (bottom right).

In addition to the genes encoding TniQ-Cascade and transposition-associated proteins, VpCAST carries pathogenesis-related genes of a type III secretion system that injects virulence proteins directly into eukaryotic host cells, thereby suppressing host immune responses [70–72]. Furthermore, other type I-F3 CAST systems from diverse bacterial species encode a variety of anti-phage defense genes, such as those involved in restriction-modification systems and cyclic oligonucleotide-based antiphage signaling systems [39, 73–75]. These observations suggest that type I-F3 CAST systems play critical roles in enabling bacteria to evade diverse biotic stresses and in disseminating survival-related defense and adaptation mechanisms across bacterial populations via horizontal gene transfer. RNA-guided DNA transposition by CAST systems represents a programmable and site-specific means of DNA integration into the genome, which is emerging as a revolutionary genome editing technology [76, 77]. Compared to the type V-K CAST systems comprising a Cas12k effector, TniQ, TnsB, and TnsC [23, 30, 37, 78], the type I-F3 CAST systems, such as VpCAST, require more protein components, thereby forming larger, more elaborate ribonucleoprotein complexes during transposition. Nevertheless, they offer advantages such as reduced off-target integration and lower rates of co-integration [21–23]. Here, we determined cryo-EM structures of VpTniQ-Cascade, which revealed its characteristic DNA recognition strategy and provided insights into the checkpoint mechanism that governs transposition initiation (Fig. 6).

A key finding of this study is the elucidation of the conformational dynamics of the TniQ-Cascade during R-loop formation, particularly involving the interplay between the Cas8/5 HB domain and the TniQ.1 HTH2 domain (Fig. 4). A recent study by Lampe *et al.* on the type I-F3 *Pseudoalteromonas agarivorans* S816 (Pse) TniQ-Cascade reported an “open” conformation, in which no interactions were observed between Cas8/5 and TniQ [79]. They further analyzed the structural dynamics using the CryoDRGN [80] algorithm, revealing an interaction between TniQ and Cas8/5 in the “closed” conformation through a low-pass filtered map. In this study, we present the VpTniQ-Cascade structures in the closed conformation across three distinct states, revealing the conformational heterogeneity at the PAM-distal site within the closed form. The cryo-EM structures of the VpTniQ-Cascade adopt three distinct states: PR, FR1, and FR2, which likely represent snapshots of sequential stages in R-loop formation, progressing from the PR state to the FR1 state and ultimately to the FR2 state (Fig. 6). Structural snapshots of the PR, FR1, and FR2 states also reveal that conformational transitions of the Cas8/5 HB domain regulate PAM-distal DNA bending through a hydrophobic interface with the TniQ.1 HTH2 domain. Disruption of this interface almost abolished transposition, highlighting its role in coordinating DNA positioning to successfully license subsequent transposition. The mobility of the Cas8/5 HB domain may enable its rearrangement during the reaction, as supported by observations of multiple cryo-EM structures showing that the local resolution of this domain is lower than that of other regions in the complex [27–29, 43, 44].

In contrast to the mismatch tolerance observed between the crRNA and the TS at the PAM-distal site in canonical type I CRISPR-Cas systems [32, 41, 42, 67], type I-F3 CAST systems strictly require correct guide-TS base pairings at this site [21, 36]. Consistently, we demonstrated that mismatches at nucleotides 25–29 and 31–32 of the crRNA–TS heteroduplex impaired the transposition efficiency (Fig. 3B). However, the molecular mechanism underlying the failure to activate transposition due to mismatches between the guide and the TS at the PAM-distal site had remained elusive, prior to this study. The present structures suggest that both the bending of the target DNA at the PAM-distal site and the orientation of the downstream DNA region toward TniQ.1, mediated by the structural rearrangement of the Cas8/5 HB domain, are crucial for transposition (Fig. 4). The establishment of correct base pairings between the crRNA and the TS at the PAM-distal site may be necessary for the precise control of this region and its distal portions. The PAM-distal region (positions 25–29) may function as a checkpoint, permitting transposition only when complete base pairing is established (Fig. 6). This notion is consistent with the observation that DNA transposition occurs at a site further downstream from the PAM-distal region [21, 81]. Based on our findings, we propose that the dynamic structural interplay between Cas8/5 and TniQ.1 serves as a molecular gatekeeper that couples DNA-binding fidelity to transposition licensing. Intriguingly, the repositioned HB domain in the FR2 state of VpTniQ-Cascade occupies a position and adopts an orientation similar to those observed in the target DNA-bound type I-F3 AsTniQ-Cascade (Fig. 4E and I) [27]. The cryo-EM structure of AsTniQ-Cascade revealed that the double-stranded region at the PAM-distal site engages the basic surface of TniQ.1, and its minor groove contacts one of the helices of the Cas8/5 HB domain (Supplementary Fig. S14). Although no dsDNA is observed at the PAM-distal site in the FR2 state of VpTniQ-Cascade, likely due to structural flexibility in this region, superimposition of the FR2 state onto AsTniQ-Cascade suggests that the HB domain is positioned in proximity to the minor groove of the dsDNA, as observed in the AsTniQ-Cascade structure. These findings suggest that, after the formation of correct base pairings at the PAM-distal site, the distorted target DNA is stabilized by the Cas8/5 HB domain and TniQ.1, a mechanism that is likely conserved among type I-F3 CAST systems.

The conformations of the crRNA at positions 33–37, which lie downstream of the guide sequence, vary among the type I-F3 TniQ-Cascade structures. Consequently, the binding modes with Cas6, Cas7.1, TniQ.1, and TniQ.2 also differ [79]. Specifically, whereas in the PseTniQ-Cascade the nucleobase at position 33 of the crRNA is located between Cas6 and TniQ.2 and oriented away from the TS, in the TniQ-Cascades from *A. salmonicida* and *V. parahaemolyticus* it is oriented toward the TS, raising the possibility that, if complementary, it might form a heteroduplex [27–29, 79]. However, introducing complementary nucleotides at positions 33–37 had no effect on the transposition efficiency, irrespective of the presence of the 8/5- and Q-loop interactions (Supplementary Fig. S12). These findings indicate that, despite the presence of a sequence complementary to the crRNA repeat in the target DNA, the heteroduplex formation does not extend beyond the guide region. Instead, they support a model in which the PAM-distal region downstream of the protospacer is required to bend the TS toward the transposase to license transposition, a process mediated by the 8/5-loop and the Q-loop.

Our structural and functional analyses revealed that type I-F3 TniQ-Cascade tolerates limited mismatches at the PAM-proximal seed region, a finding that contrasts with canonical type I CRISPR-Cas systems [41, 42, 62, 63]. This mismatch tolerance may correlate with the structural plasticity of the heteroduplex-binding channel, which is likely mediated by the absence of a “lid-like” residue, such as Arg85 of Cas5 in type I-F1 PaCascade that stabilizes the PAM-proximal base pairs [18], thereby reducing the energetic penalty for accommodating non-Watson–Crick geometries at the seed region. Consistent with this, the observed Hoogsteen and A-C mismatches fit snugly in the channel of VpTniQ-Cascade and do not abolish the activity. We also found that cumulative mismatches still suppress transposition. Collectively, while the seed region in type I-F3 systems remains essential for transposition, it appears to tolerate more mismatches than the type I-F1 systems responsible for adaptive immunity [41, 42, 62, 63]. Biologically, PAM-proximal mismatch-induced reductions in transposition efficiency may serve as an additional regulatory mechanism for CAST transposition, complementing the transcriptional control by Xre and the intrinsically modest catalytic activity of the TnsB integrase [26, 77, 79], and thereby modulating the transposition frequency and limiting unintended genomic damage. Notably, bacteria encoding both type I-F1 and I-F3 systems have been described, and the type I-F1 Cascade can use canonical crRNAs produced by I-F3a and I-F3b systems, as well as atypical crRNAs generated by I-F3a CAST [26]. The type I-F1 Cascade containing such crRNAs from I-F3 CAST is active, potentially enabling the targeting of genomic DNA or resident plasmids [26]. In this context, the mismatch-induced reduction in transposition efficiency observed here may serve as a safeguard against I-F1-mediated self-targeting and subsequent Cas2/3-mediated degradation. Although mismatches between atypical crRNAs and *att* sites are common and unlikely to affect CAST homing into genomic DNA, to our knowledge, this is the first proposal that the transposition efficiency can be modulated by canonical crRNAs bearing mismatched spacers.

VpTniQ-Cascade preferentially recognizes the 5′-CN-3′ PAM (Fig. 2B). Compared to other type I-F3 CAST systems, such as those from *V. cholerae* and *A. salmonicida* [27, 39, 81], the PAM preference of VpTniQ-Cascade appears to be more restricted. Notably, the type I-F3 AsCAST has a broad PAM preference spectrum. It recognizes thymine at position −2 of the PAM with an efficiency comparable to that for cytidine. AsCAST uses the *ffs* gene as the *att* site for transposition [26, 27]. When the type I-F3 CAST utilizes the *ffs* gene as the *att* site, the 5′-TC-3′ PAM is predominantly used (96.6%) [27]. This observation suggests that the thymine preference at position −2 of the PAM is essential for efficient transposition into the genome. In contrast, all type I-F3 CAST systems that use the *yciA* gene as the *att* site recognize 5′-CA-3′ or 5′-CG-3′ PAMs [21, 26], consistent with the PAM preference of VpTniQ-Cascade. These findings suggest that the PAM preferences among type I-F3 CAST systems may have co-evolved with the *att* site selection to ensure reliable transposition. Sequence and structural analyses indicate that Asp126 in Cas8/5 plays a central role in determining the PAM specificity of VpTniQ-Cascade by mediating a hydrogen bond with dG(−2) complementary to dC(−2)* of the 5′-CN-3′ PAM. The conservation of this residue as either Asp or Asn across type I-F Cascade suggests its evolutionary importance in PAM recognition. Notably, the mutation of Asp126 to alanine markedly broadened the PAM preference, underscoring its role as a molecular determinant of cytidine selectivity. These findings provide mechanistic insight into the structural basis for PAM recognition in type I-F3 CAST systems and suggest a strategy for tuning PAM recognition for genome editing applications. In contrast to the stringent PAM recognition observed in type I-F1 Cascade involved in adaptive immunity [19, 82], CAST systems catalyze DNA integration and therefore appear to tolerate broader PAM ranges. The more relaxed PAM requirements in CAST systems may serve as an evolutionary adaptation that expands the pool of potential integration sites, facilitating transposon mobility. By contrast, host-genome homing is restricted to a small set of *att* sites, in which 94% of the events occur at four loci (*yciA, ffs, guaC*, and *rsmJ* genes), suggesting that the acquisition of new spacers for genomic integration is limited, as previously proposed [26]. Among these genes, *ffs, guaC*, and *rsmJ* are essential, whereas *yciA* is not. The fact that the *yciA att* site is the most prevalent (56%) in type I-F3 CAST systems suggests that integration near *yciA* may represent the safest option among the four loci. In other words, accidental disruption of *yciA* would be less detrimental to cellular viability. This rationale may help to explain the high prevalence of *yciA* as an *att* site.

We demonstrated that VpCAST enables functional gene integration in a physiologically relevant context (Fig. 5). Using a methionine auxotrophic *E. coli* strain [68, 69], we successfully restored cell growth under methionine-deficient conditions through CAST-mediated insertion of the intact *metE* gene. This system also offers a growth-based selection platform that can serve for screening enhanced CAST variants; for example, through random mutagenesis of components of TniQ-Cascade as well as transposition-associated proteins. Recently, the phage-assisted continuous evolution (PACE) technique was applied to generate CAST variants with substantially improved integration activity in human cells, enabling the site-specific insertion of DNA fragments up to 15 kbp in size [77]. In contrast to the PACE-based evolution of CAST, our approach may offer a more facile method for screening functional CAST variants generated by error-prone PCR, as well as for evaluating the functions of amino acid residues in each component. Collectively, this study has shown that the modularity and programmability of VpCAST, combined with its demonstrated structural plasticity and engineering potential, underscore its value as a next-generation tool for developing precise, safe, and customizable genome editors.

## Supplementary Material

gkaf1415_Supplemental_Files

## Data Availability

The atomic coordinates of VpTniQ-Cascade in the PR state, FR1 state, and FR2 state have been deposited in the Protein Data Bank under accession codes 9VTP, 9VTQ, and 9VTR, respectively. The associated cryo-EM maps are available in the Electron Microscopy Data Bank under accession codes EMD-65338, EMD-65339, and EMD-65340, respectively.
