# Supplementary material for: Sequential structural rearrangements at the PAM-distal site of a type I-F3 CRISPR-Cas effector enabling RNA-guided DNA transposition
Source: Nucleic Acids Res. 2026 Jan 6;54(1):gkaf1415. doi: 10.1093/nar/gkaf1415 (PMC12774636; doi:10.1093/nar/gkaf1415)
Supplement: gkaf1415_Supplemental_Files [file gkaf1415_supplemental_files.zip › Supplementary_Fig_20251121_TIFF.pdf]

Supplemental information

Sequential structural rearrangements at the PAM-distal site of a type I-F3 CRISPR-Cas effector enabling RNA-guided DNA transposition

Kazuki Ishihara, Shunsuke Matsumoto, Christoph Gerle, Chai C. Gopalasingam, Tsuyoshi Shirai, Hideki Shigematsu, and Tomoyuki Numata

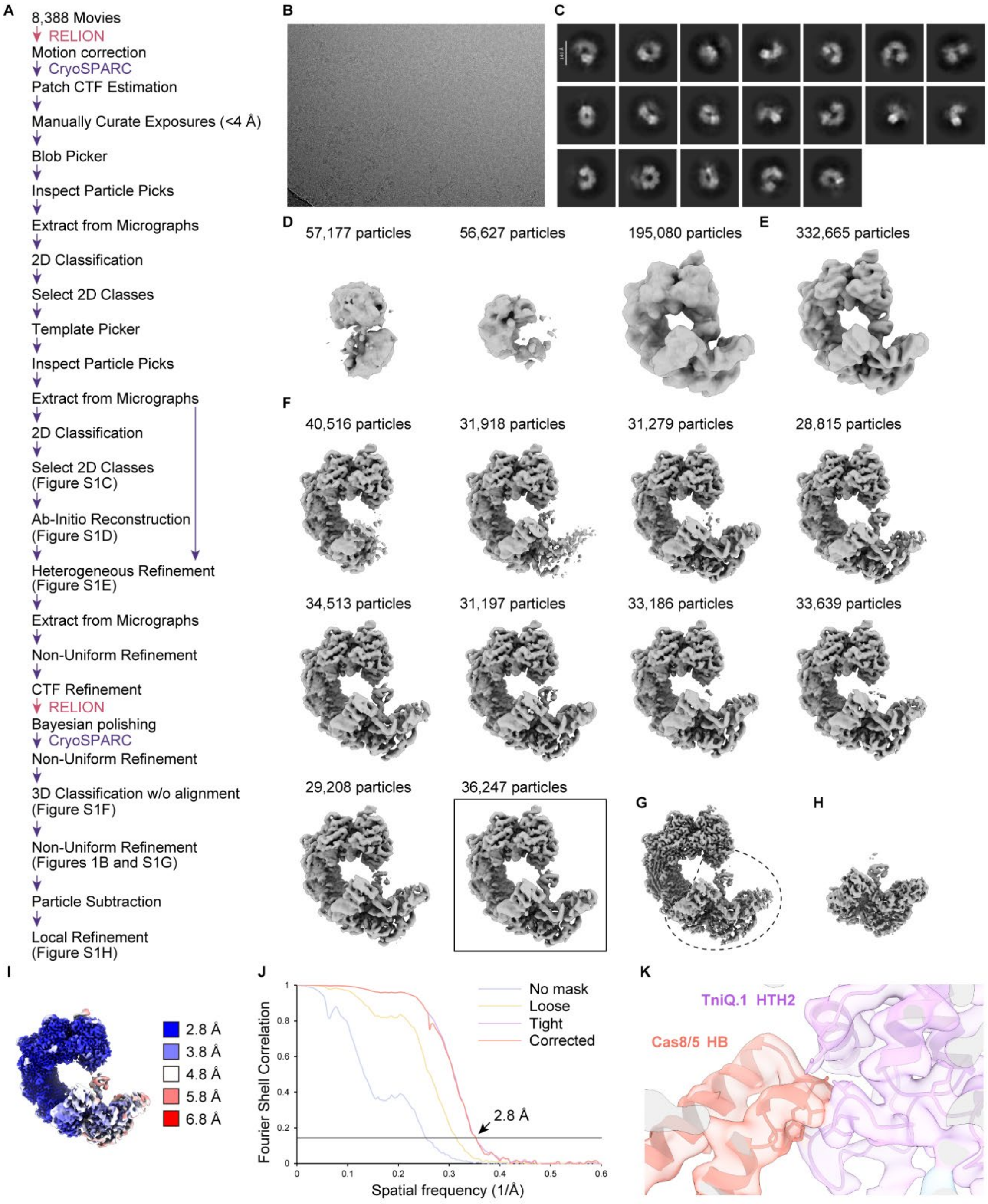

Supplementary Figure S1. Single-particle analysis of VpTniQ-Cascade in form 1 (FR1 state).

(A) Workflow of single-particle analysis.

(B) Representative motion-corrected micrograph.

(C) Selected 2D class-averages for Ab-Initio Reconstruction.

(D) Maps from Ab-Initio Reconstruction.

(E) 3D maps classified by multi-reference refinement through heterogeneous refinement.
(F) Maps classified by 3D classification without alignment in CryoSPARC. The 3D map enclosed in the box denotes the class selected for further analysis.
(G) The 3D Map refined by Non-Uniform Refinement in CryoSPARC. Dashed lines indicate the region of the focused mask generated for the subsequent job.
(H) Focused map of the 3′ end of the crRNA, Cas7.1, Cas8/5 HB domain, Cas6, and the two TniQ molecules.
(I) Estimated local resolution colored on the map.
(J) Gold-standard Fourier shell correlation (FSC) curve. The solid horizontal line indicates the FSC at 0.143.
(K) Model fitted into the focused map of the Cas8/5 HB and TniQ.1 HTH2 regions.

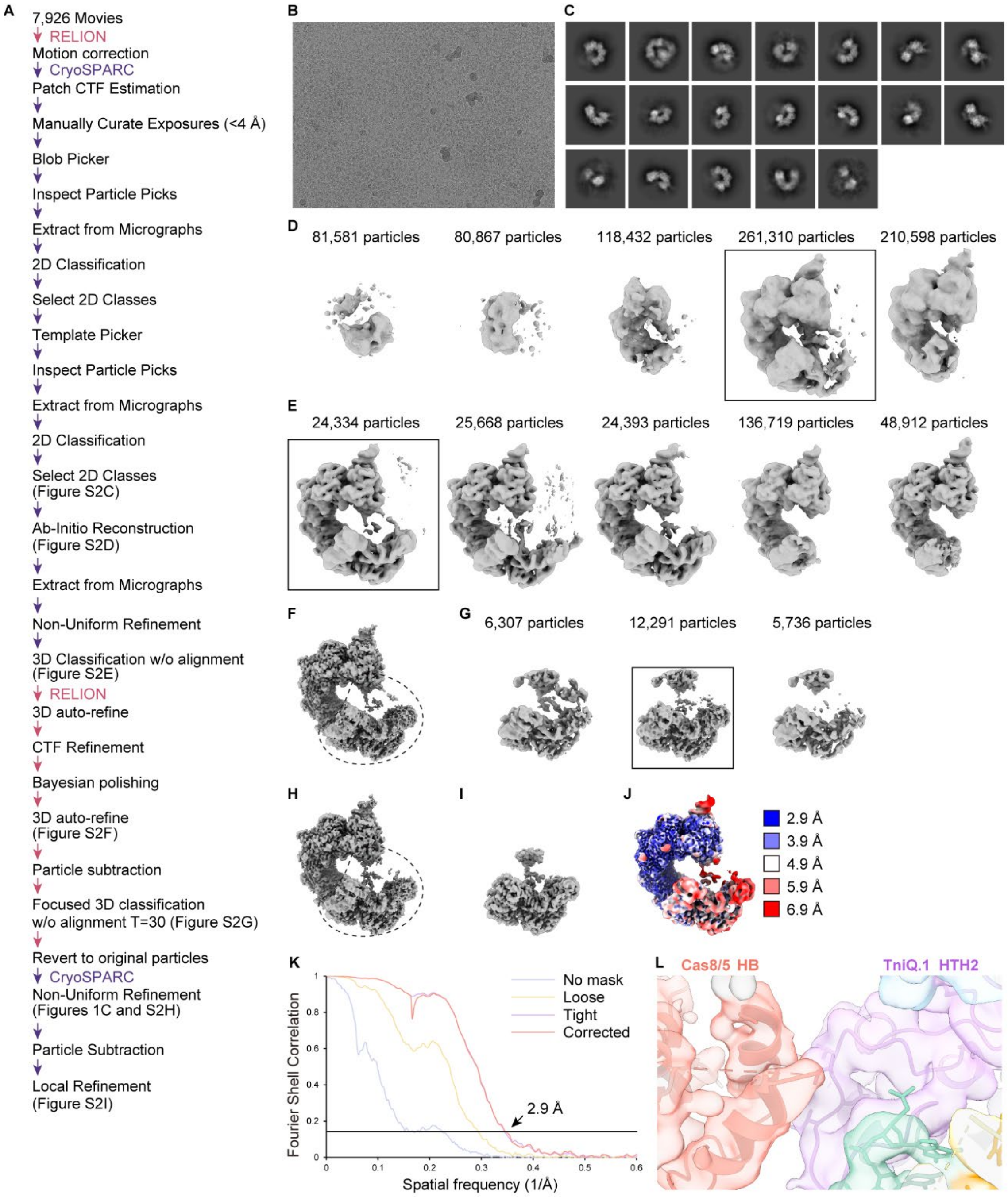

Supplementary Figure S2. Single-particle analysis of VpTniQ-Cascade in form 2 (FR2 state).

(A) Workflow of single-particle analysis.

(B) Representative motion-corrected micrograph.

(C) Selected 2D class-averages for Ab-Initio Reconstruction.

(D) Maps from Ab-Initio Reconstruction. The 3D map enclosed in the box indicates the

class selected for subsequent analysis.
(E) Maps classified by 3D classification without alignment in CryoSPARC. The 3D map enclosed in the box denotes the class selected for further analysis.
(F) The 3D Map refined by 3D auto-refine in RELION. Dashed lines surround the region of the focused mask generated for the subsequent job.
(G) Maps classified by focused 3D classification without alignment in RELION. The 3D map enclosed in the box indicates the class selected for further analysis.
(H) The 3D Map refined by Non-Uniform Refinement in CryoSPARC. Dashed lines surround the region of the focused mask generated for the subsequent job.
(I) Focused map of the 3′ end of the crRNA, Cas7.1, Cas8/5 HB domain, Cas6, and the two TniQ molecules.
(J) Estimated local resolution colored on the map.
(K) Gold-standard Fourier shell correlation (FSC) curve. The solid horizontal line indicates the FSC at 0.143.
(L) Model fitted to the focused map sharpened by DeepEMhancer of the Cas8/5 HB and TniQ.1 HTH2 regions.

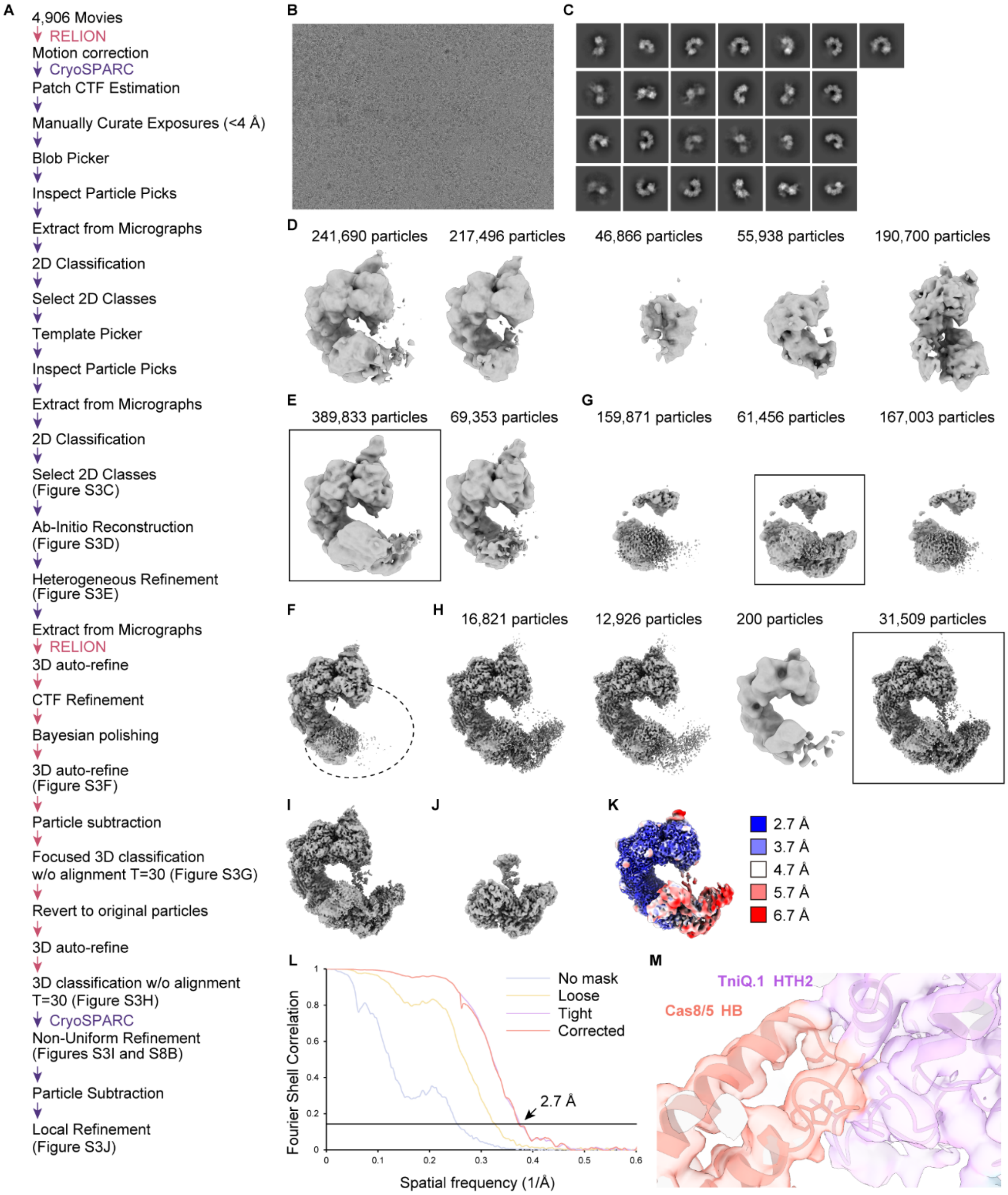

Supplementary Figure S3. Single-particle analysis of VpTniQ-Cascade complexed with the *hflK* protospacer (PR state).

(A) Workflow of single-particle analysis.

(B) Representative motion-corrected micrograph.

(C) Selected 2D class-averages for Ab-Initio Reconstruction.

(D) Maps from Ab-Initio Reconstruction.

(E) 3D maps classified by multi-reference refinement through heterogeneous refinement. The 3D map enclosed in a box denotes the class selected for further analysis.
(F) 3D Map refined by 3D auto-refine in RELION. Dashed lines surround the region of the focused mask generated for the subsequent job.
(G) Maps classified by focused 3D classification without alignment in RELION. The 3D map enclosed in a box indicates the class selected for subsequent analysis.
(H) VpTniQ-Cascade classified in RELION after signal restoration of the original particles. The 3D map enclosed in a box indicates the class selected for further analysis.
(I) Final map of VpTniQ-Cascade complexed with the *hflK* protospacer.
(J) Focused map of the 3′ end of the crRNA, Cas7.1, Cas8/5 HB domain, Cas6, and the two TniQ molecules.
(K) Estimated local resolution colored on the map.
(L) Gold-standard Fourier shell correlation (FSC) curve. The solid horizontal line indicates the FSC at 0.143.
(M) Model fitted into the focused map sharpened by DeepEMhancer of the Cas8/5 HB and TniQ.1 HTH2 regions.

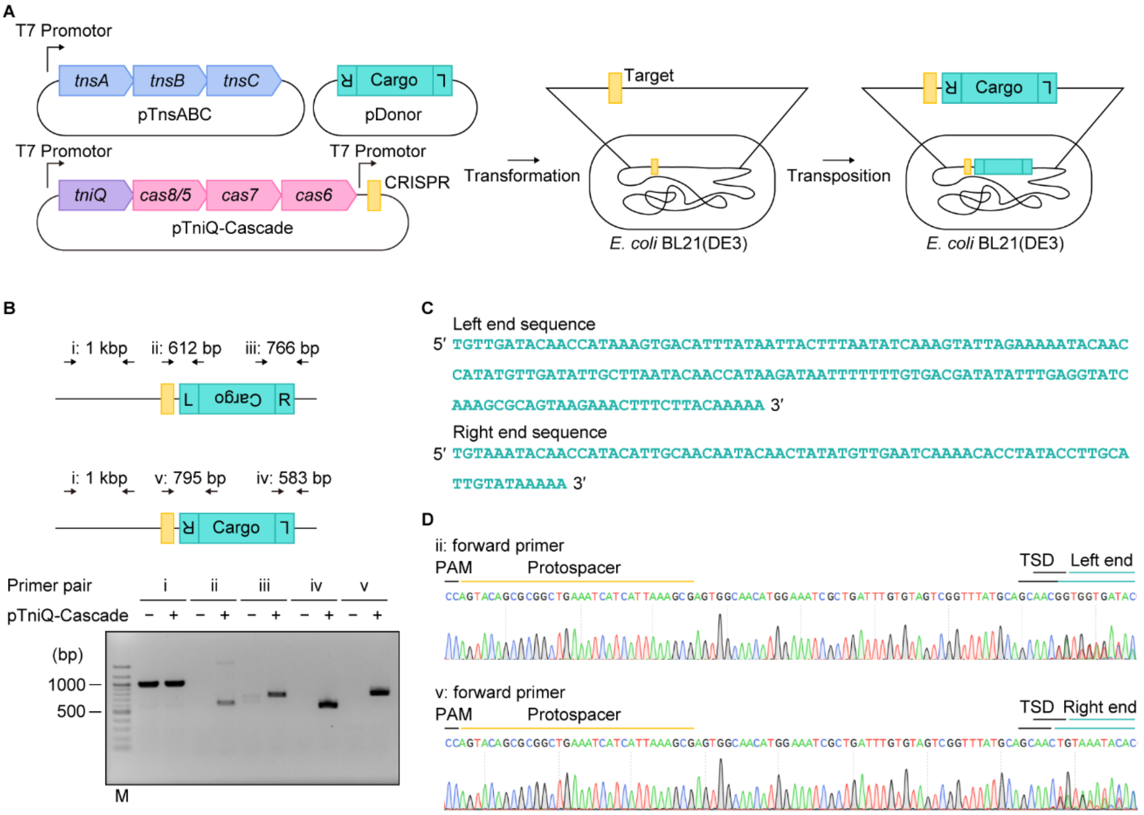

Supplementary Figure S4. *In vivo* reconstitution of transposition into the genomic DNA of *E. coli* strain BL21(DE3).

(A) Schematic diagram of the *in vivo* transposition experiments. The CRISPR array containing a single spacer targeting the sequence of the *lacZ* gene on pTniQ-Cascade and the corresponding target sequence in the genomic DNA of *E. coli* strain BL21(DE3) are shown in light yellow.

(B) Schematic representation of transposition into the genomic DNA of *E. coli* cells in both forward and reverse orientations (top). The positions of primer pairs and the sizes of the PCR amplicons are indicated above the corresponding regions. The amplicons were analyzed by electrophoresis on a 1.5% agarose gel (bottom). The gel image is representative of three independent experiments.

(C) Terminal sequences of VpCAST. The sequences of the left end (top) and right end (bottom) are shown.

(D) Determination of transposition sites. Electropherograms obtained by Sanger sequencing of PCR amplicons using primer pairs ii (top) and v (bottom) are shown. The PAM sequence, protospacer sequence, TSD (target site duplication), and left-end and right-end sequences of VpCAST are highlighted.

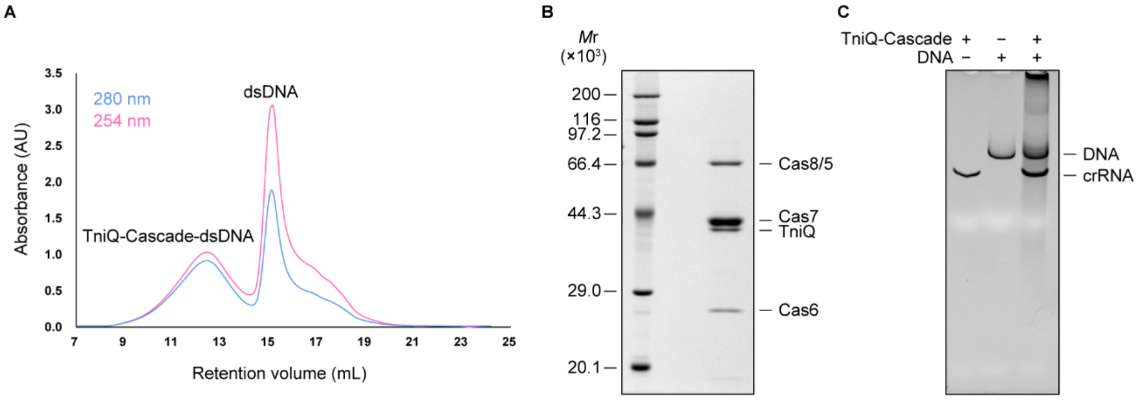

Supplementary Figure S5. Purification of dsDNA-bound VpTniQ-Cascade.

(A) Size-exclusion chromatography analysis of dsDNA-bound VpTniQ-Cascade. The x-axis represents the retention volume, and the y-axis indicates absorbance.

(B) SDS-PAGE analysis of dsDNA-bound VpTniQ-Cascade. Proteins were separated on a 12% polyacrylamide gel containing SDS.

(C) Denaturing PAGE analysis of dsDNA-bound VpTniQ-Cascade. Samples were separated on a 15% polyacrylamide gel containing 8 M urea.

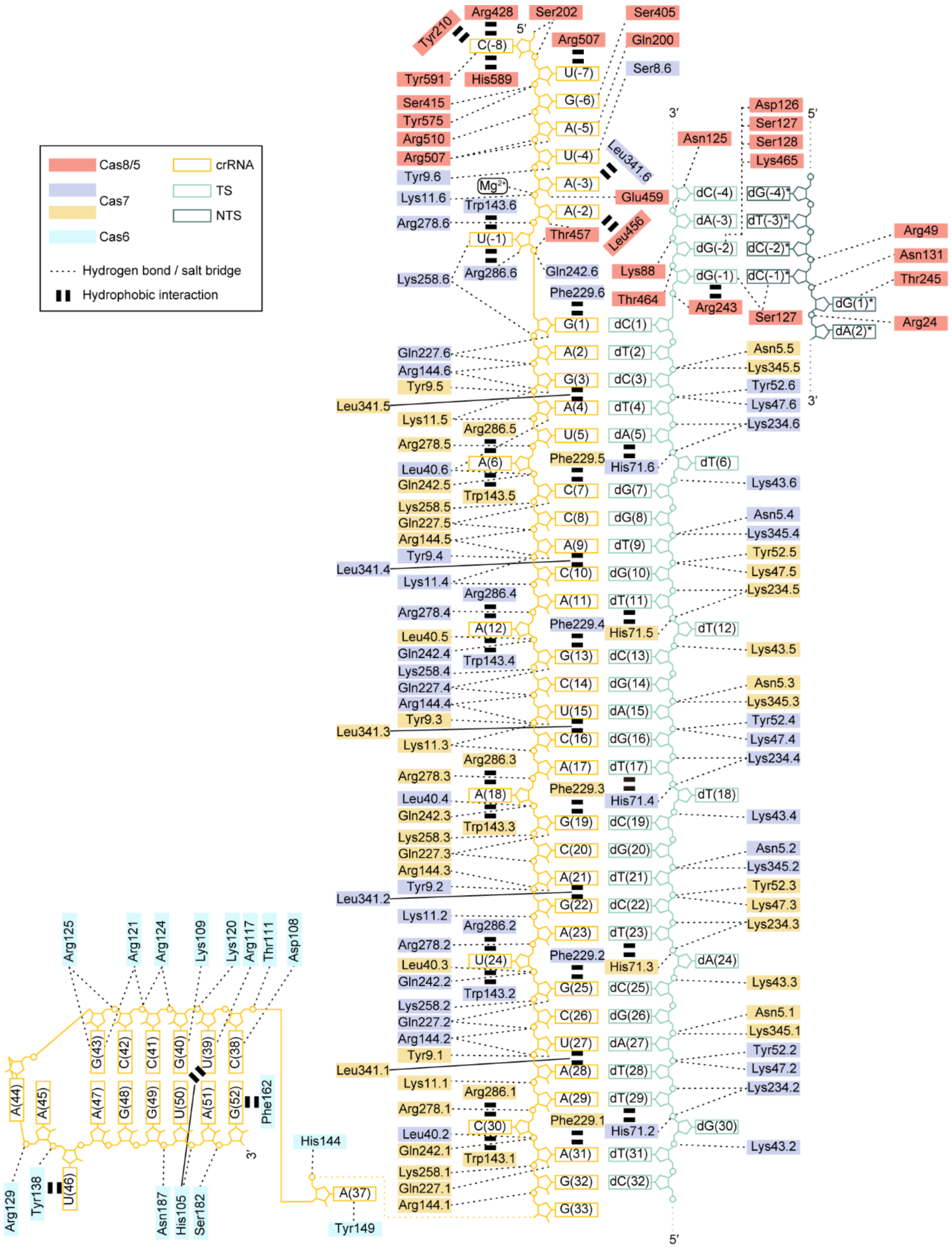

Supplementary Figure S6. Schematic diagram of interactions between nucleic acids and protein subunits.

For Cas7, subunit numbers are indicated following the residue numbers. Thin and thick black dashed lines represent hydrogen bonds and stacking interactions, respectively.

Disordered regions of nucleic acids are shown as dashed lines in their respective colors.

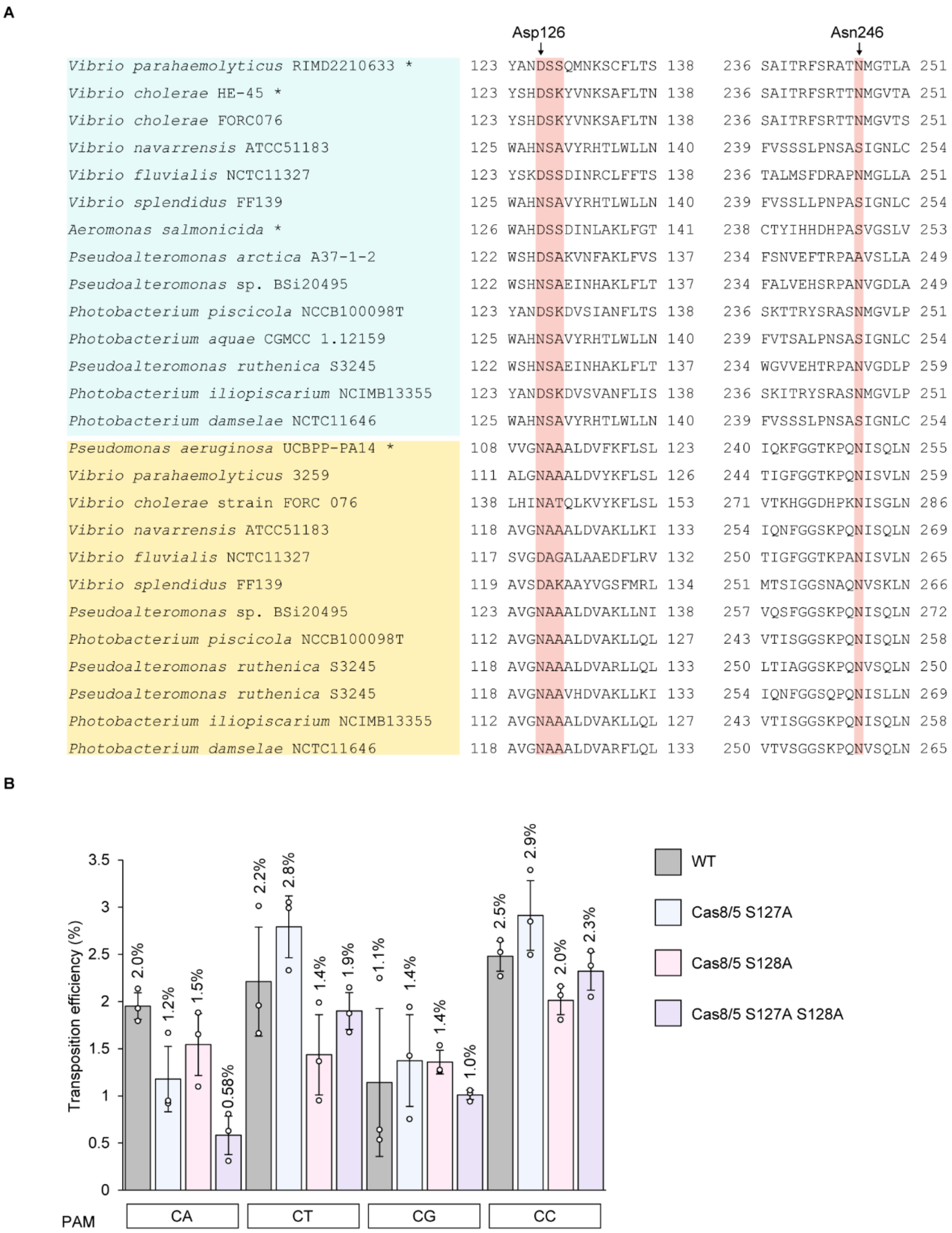

Supplementary Figure S7. PAM-interacting residues in type I-F3 Cas8/5 and type I-F1 Cas8.

(A) Amino acid sequence alignments surrounding Asp126 and Asn246 of VpCas8/5 are shown. Residue numbers are indicated at both ends of each sequence. Type I-F3 Cas8/5 and type I-F1 Cas8 are highlighted in light cyan and light yellow, respectively. Species

marked with an asterisk indicate those for which three-dimensional structures have been determined to date. Asp126, Ser127, Ser128 and Asn246 in VpCas8/5 and their corresponding residues are highlighted in light coral.

(B) *In vivo* transposition assay of wild-type VpTniQ-Cascade, Cas8/5 S127A, Cas8/5 S128A, and the double mutant-containing VpTniQ-Cascade for 4 different PAM sequences. Transposition efficiencies, evaluated by qPCR, are plotted. Data are presented as mean ± SD from three independent experiments. The mean transposition efficiency is indicated above each bar.

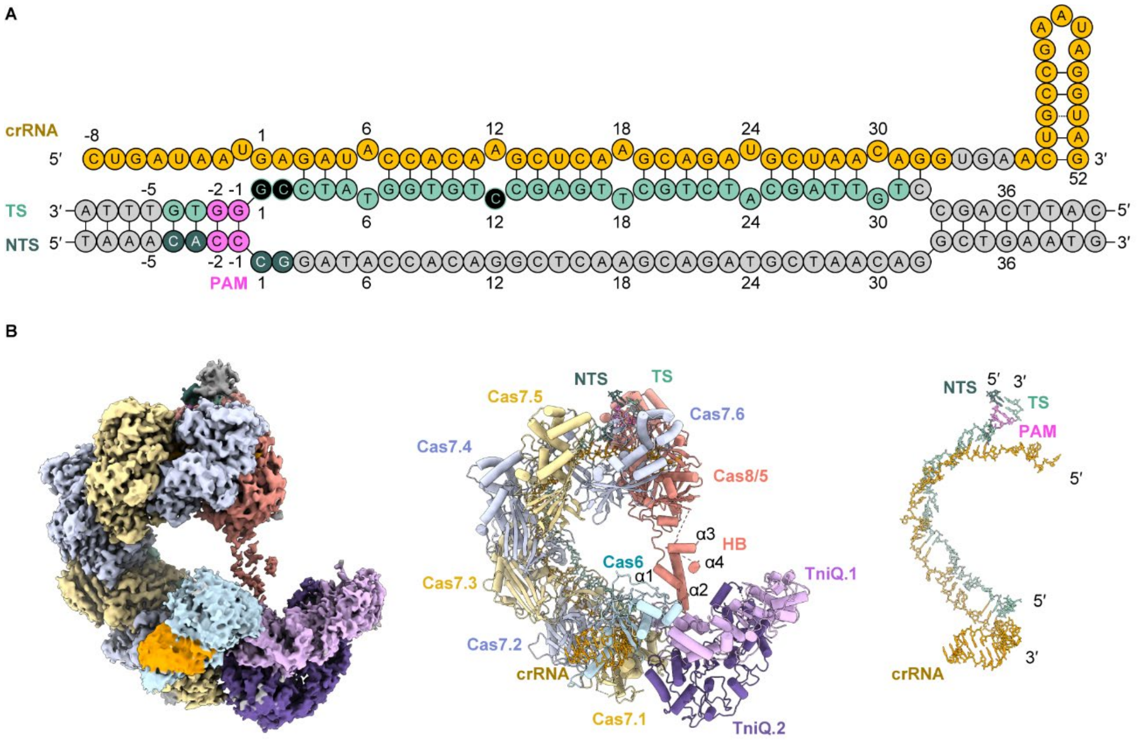

Supplementary Figure S8. The *hflK* gene harboring a putative protospacer targeted by the first spacer of VpCAST.

(A) Schematic diagram of the R-loop formed between the crRNA derived from the first spacer of the CRISPR array and the target DNA containing the *hflK* protospacer, which results in mismatches at positions 1, 2, and 12. The crRNA, TS, and NTS are colored yellow, light green, and dark green, respectively. The PAM sequence is colored pink. TS nucleotides at positions 1, 2, and 12 that are mismatched with the crRNA are colored black. Disordered nucleotides in the cryo-EM structure are shown in gray. Nucleotide numberings for each strand are indicated.

(B) Overall structure of VpTniQ-Cascade bound to dsDNA containing the *hflK* protospacer, determined by cryo-EM single particle analysis. (Left) Cryo-EM map of the complex. (Middle) Cartoon representation of the complex. The numbering of α-helices in the Cas8/5 HB domain is indicated. (Right) Stick representations of the bound target DNA and crRNA within the complex. Color codes for each protein subunit and nucleic acid are consistent with those used in the main Figure 1.

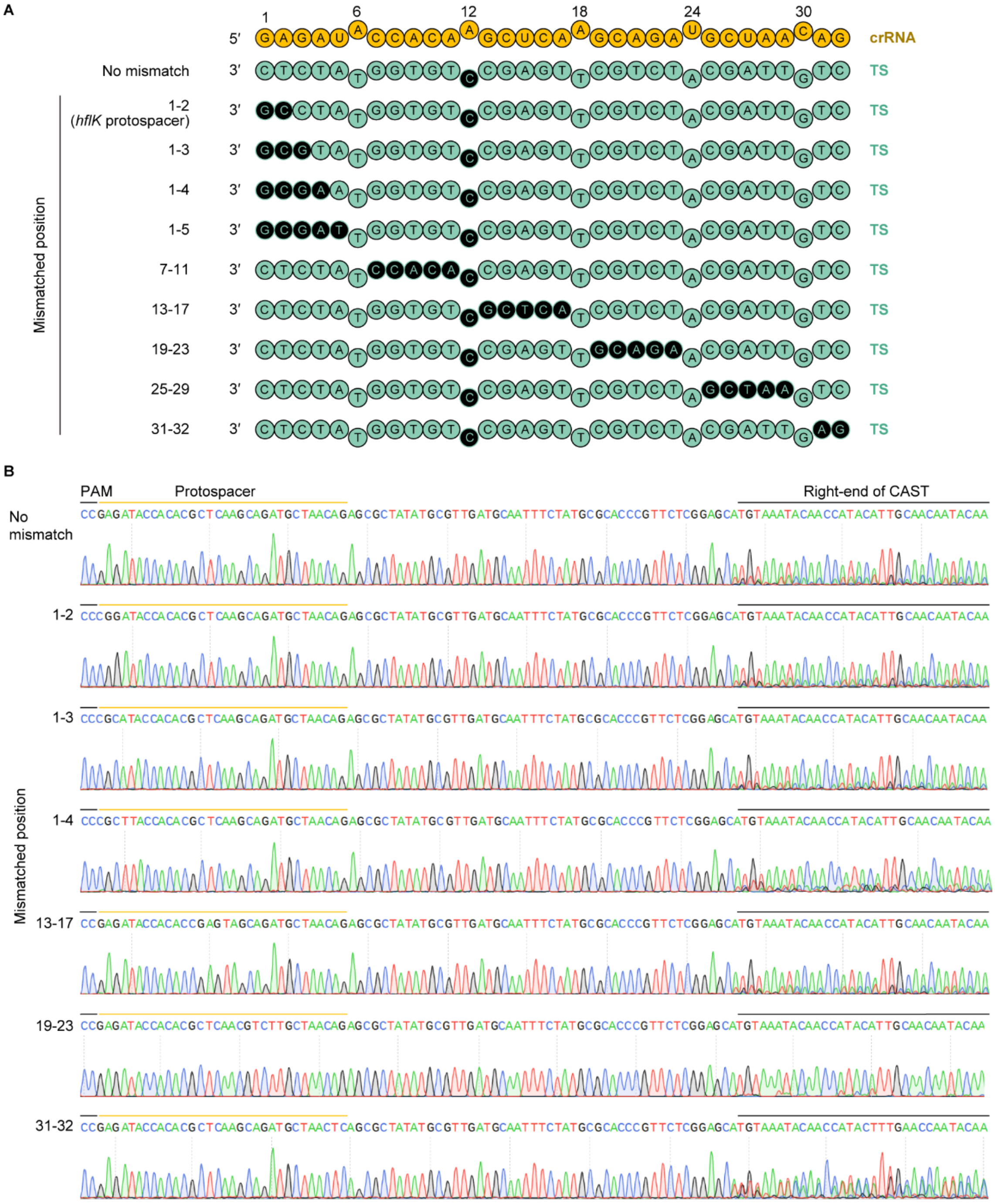

Supplementary Figure S9. Transposition into target DNA containing mismatches in various nucleotide positions.

(A) Design of target DNA containing mismatches within the protospacer. Only the sequences of the TS are shown; the NTS sequences are omitted. The crRNA and TS are colored yellow and light green, respectively. TS nucleotides that are mismatched with the crRNA are colored black.

(B) Determination of transposition sites. Electropherograms obtained from Sanger sequencing of PCR amplicons are shown. The mismatched positions of each construct are indicated on the left. The PAM sequence, the protospacer sequence, and the right-end sequence of VpCAST are highlighted.

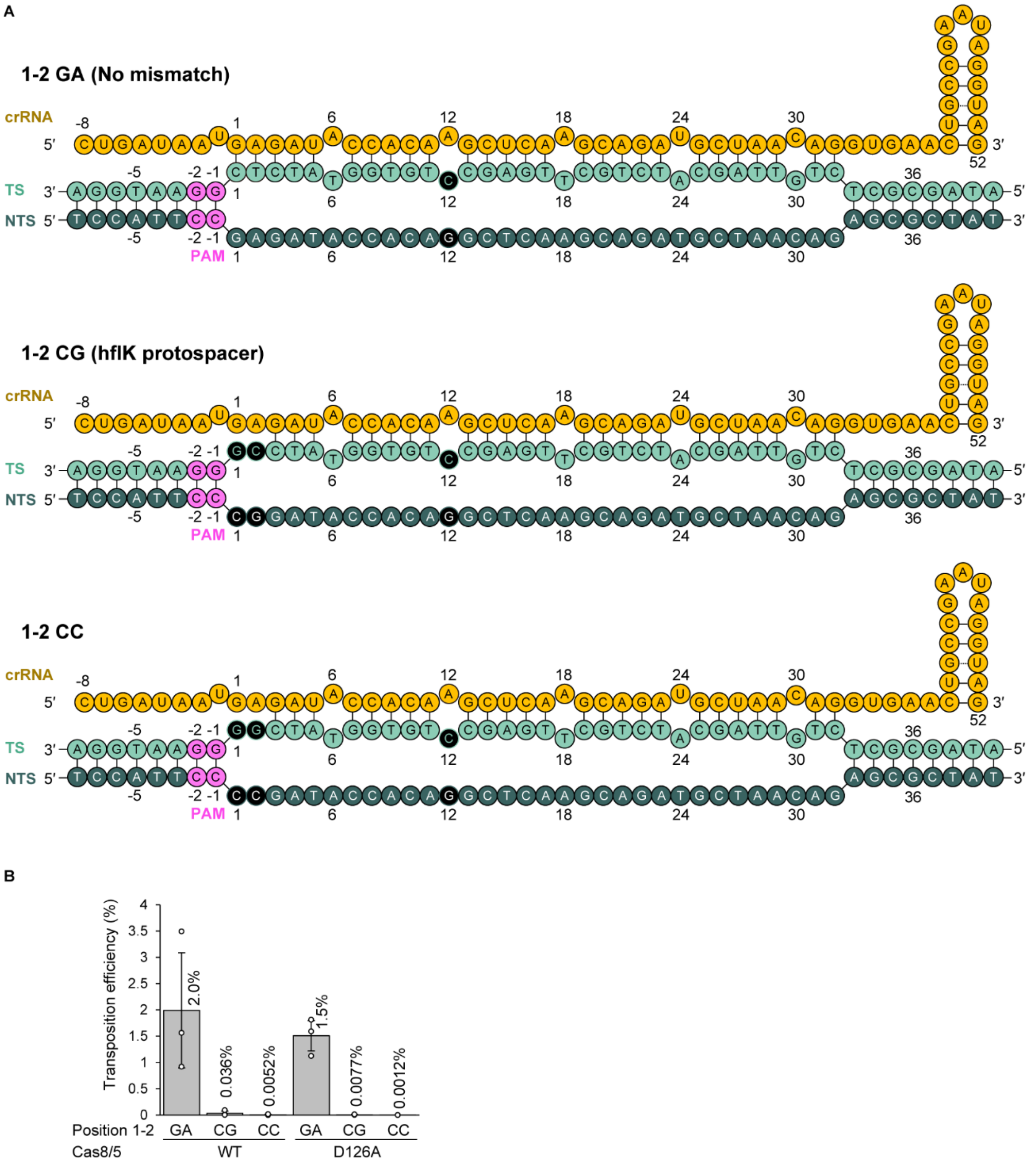

Supplementary Figure S10. Transposition into target DNA containing mismatches at positions 1–2.

(A) Schematic diagrams of the R-loop structures formed between the crRNA and target DNA. The crRNA-TS heteroduplex without mismatches in any of the 5-nt segments (top), with mismatches at positions 1–2 bearing a CG sequence (middle; native *hflK* protospacer), and with mismatches at positions 1–2 bearing a CC sequence (bottom). The crRNA, TS, and NTS are colored yellow, light green, and dark green, respectively. Mismatched nucleotides are colored black. The PAM sequence is pink. Nucleotide

numberings for each strand are indicated.

(B) *In vivo* transposition efficiencies for matched and mismatched target DNAs in the presence of wild-type or Cas8/5 D126A mutant TniQ-Cascade. Transposition efficiencies determined by qPCR are plotted. Data are presented as mean ± SD from three independent experiments.

A

Thr457 ↓

Vibrio parahaemolyticus RIMD2210633 *	437	FKIESFAVCIHNIHVENRGLTREWVPNITAPATRDDWQCDV	481
Vibrio cholerae HE-45 *	433	FRVESFAICVHQLHVEKRGLTAEFVEKISAPATRDDWQCDV	477
Vibrio cholerae FORC076	433	FRVESFAICVHQLHVEKRGLTTEFVEKISAPATRDDWQCDV	477
Vibrio navarrensis ATCC51183	483	VEFSSFAFYVRNENIQSTAKLTEPNSVAKRPTIRSERLADL	531
Vibrio fluvialis NCTC11327	437	FTIDSFAICIHNYHLEKRGLTKEAIKKIAPPAIYDDWQFDS	487
Vibrio splendidus FF139	483	FEFSSFSFYVRSENIQPTAKLTEPNSVAKRPTIRSERLADL	531
Aeromonas salmonicida *	486	VYFRGLAWYLGRYSLVTGKHLPEPSKSIRRPGLLDGRYCDL	534
Pseudoalteromonas arctica A37-1-2	483	VRFTSFSWFIRDYSAVAGKKLPELSLQLKRPGIIDGKYCDL	529
Pseudoalteromonas sp. BSi20495	465	IKCIGSAIYIENYQLYTGKPLPEPSKLVISSGIIDKPKCDI	513
Photobacterium piscicola NCCB100098T	437	FKVKSFAICIHTFHLQKRGMTRESVEKISPPATHDEWYCDL	481
Photobacterium aquae CGMCC 1.12159	483	FEFSRFAFYVRTENIQSTAKLTEPNSLAKRPTIRSERLADL	531
Pseudoalteromonas ruthenica S3245	468	IECIGSAIFVHSYQLHTGKPLPEQNQLAKRPGIVDKPKCDM	516
Photobacterium iliopiscarium NCIMB13355	433	LKVDSFAICIHTFHLQKRGMTRESIIKISSPATHDEWHCDL	477
Photobacterium damselae NCTC11646	483	FEFSSFSFYIRSERIQLTAKLTEPNSVAKRPTIRSELLADL	531
Pseudomonas aeruginosa UCBPP-PA14 *	65	RFEAQISQPAGKRTKVFNLTRNPLNRDGSTAAIVEEGRAHL	105
Vibrio parahaemolyticus 3259	34	QFDLQTHKGEGDFVHSIIGTGNPLDKDGSRSAFIEEARCHL	74
Vibrio cholerae strain FORC 076	60	RCDVQTHKGEGDFVNSILGTRNPSVFDLTSPSFIEEARCHL	118
Vibrio navarrensis ATCC51183	59	KNQVHARQPKGWGDFVFALTRNPLTHQGKTAPINEEGRMNM	99
Vibrio fluvialis NCTC11327	61	RCDVQTHKGEGDFVHSIIGTGNPLDKDGSRSAFIEEARCHL	101
Vibrio splendidus FF139	60	QHQVHAYRENQYEPYSFALTRNPLTKEGKTAPINEEGRMHL	100
Pseudoalteromonas sp. BSi20495	28	KNIVHARQPKGWGDYVFALSRNPLTHQGKTAPINEEGRMHM	68
Photobacterium piscicola NCCB100098T	58	QYQVHAYRGSDWDDYGFALTRNPLAKDGKTAPIVEEGKMNL	98
Pseudoalteromonas ruthenica S3245	60	QHQVHAYRESIYEPYSFALTRNPLTKEGKTAPINEEGRMHL	100
Pseudoalteromonas ruthenica S3245	59	KNQVHARQPKGWGDYVFALTRNPLTYQGKTAPINEEGRMNM	99
Photobacterium iliopiscarium NCIMB13355	58	QHQVHAYRGSDWDDYGFSLTRNPLAKDGKTAPIVEEGKMNL	98
Photobacterium damselae NCTC11646	58	QHQTHAYRESSYEPYSFALTRNPLTKEGKTAPINEEGRMSM	98

B

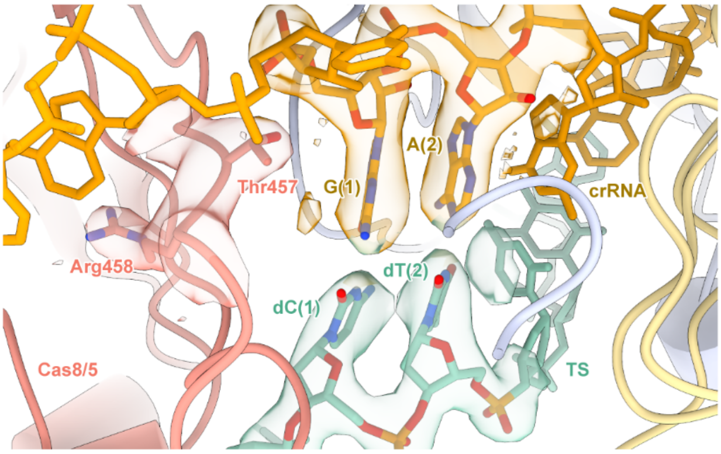

Supplementary Figure S11. Comparison of amino acid residues between the type I-F3 and type I-F1 Cascade located near nucleotide positions 1 and 2 within the seed region. (A) Amino acid sequence alignments of type I-F3 Cas8/5 and type I-F1 Cas5. The region surrounding Thr457 in VpCas8/5 and the corresponding residue, Arg85, in PaCas5 is shown. Residue numbers are indicated at both ends of each sequence. Type I-F3 Cas8/5

and type I-F1 Cas5 are highlighted in light cyan and light yellow, respectively. Species marked with an asterisk indicate those for which three-dimensional structures have been determined to date. Residues corresponding to Thr457 in VpCas8/5 and Arg85 in PaCas5 are highlighted in light coral.

(B) Close-up view of the seed region of VpTniQ-Cascade bound to the *hflK* protospacer. Protein subunits and nucleic acids are depicted as cartoon and stick models, respectively. Thr457 and Arg458 of VpCas8/5 are also shown in stick models. It is noteworthy that VpCas8/5 Arg458 does not function as a lid, in contrast to PaCas5 Arg85. The cryo-EM map around the nucleotides at positions 1 and 2 is displayed.

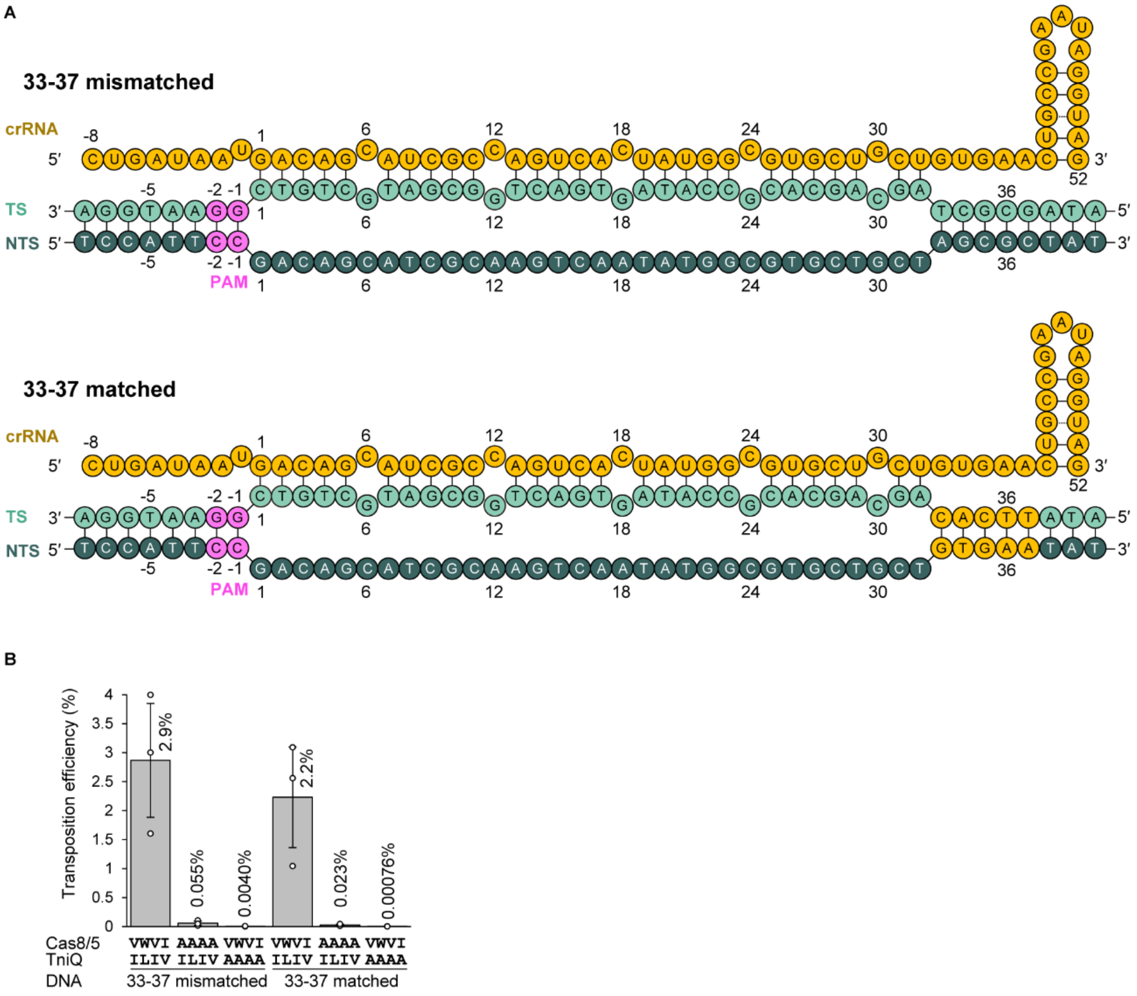

Supplementary Figure S12. Transposition into target DNA containing nucleotides complementary to positions 33–37 of the crRNA 3′ repeat.

(A) Schematic diagrams of the R-loop structures formed between the crRNA and target DNA lacking or containing complementarity at positions 33–37 (top and bottom, respectively). The crRNA, TS, and NTS are colored yellow, light green, and dark green, respectively. Nucleotides in the TS and the corresponding NTS that are complementary to positions 33–37 of the crRNA 3′ repeat are highlighted in yellow. The PAM sequence is pink. Nucleotide numbering of each strand is indicated.

(B) *In vivo* transposition efficiencies for target DNA containing nucleotides complementary to positions 33–37 of the crRNA 3′ repeat in the presence of wild-type, 8/5-loop, or Q-loop mutants. Transposition efficiencies determined by qPCR are plotted. Data are presented as mean ± SD from three independent experiments.

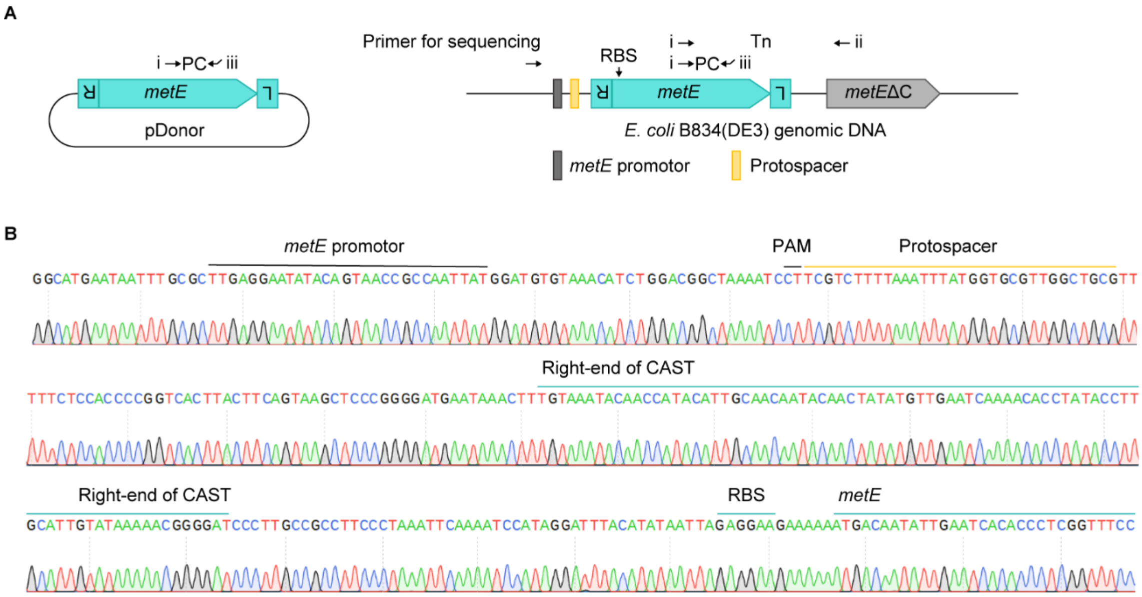

Supplementary Figure S13. Transposition sites of *metE* gene in *E. coli* strain B834(DE3).

(A) Schematic diagram showing the annealing positions of primers used for PCR and Sanger sequencing.

(B) Transposition site of the *metE* gene, determined by Sanger sequencing. The electropherogram of the PCR amplicon using the transposed sample is shown. The sequences of the *metE* promotor, PAM, protospacer, ribosome binding site (RBS), *metE* gene, and the right-end of VpCAST are indicated.

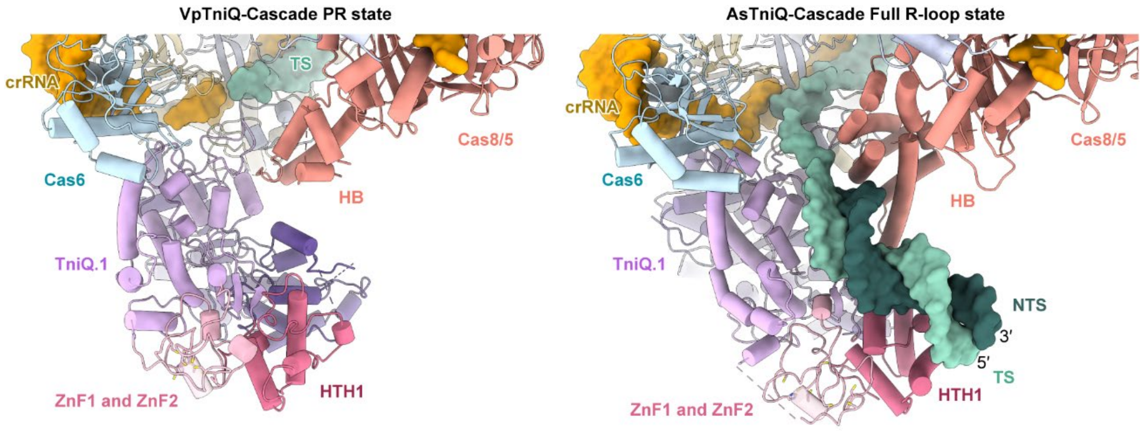

Supplementary Figure S14. dsDNA binding region downstream of the PAM-distal site. Structural comparison of the dsDNA-binding regions downstream of the PAM-distal site of VpTniQ-Cascade in the PR state (left) and AsTniQ-Cascade (right). The ZnF (zinc finger) and HTH1 (helix-turn-helix 1) domains of TniQ.1 are shown in light pink and pink, respectively. The crRNA and target DNA are represented as surface models.
